# The Effectiveness of Blood Flow Restriction Training on Rehabilitation After Anterior Cruciate Ligament Reconstruction: A Systematic Review and Meta-Analysis

**DOI:** 10.3390/jcm15124706

**Published:** 2026-06-17

**Authors:** Nan Xu, Tingting Wang, Ruibin Guo, Yinglu Hong, Vilma Dudonienė

**Affiliations:** 1 Department of Health Promotion and Rehabilitation, Lithuanian Sports University, Sporto g. 6, 44421 Kaunas, Lithuania; nan.xu@stud.lsu.lt; 2 School of Strength and Conditioning Training, Beijing Sport University, Xinxi Road No. 48, Beijing 100084, China; 2023210118@bsu.edu.cn (T.W.); guoruibin@bsu.edu.cn (R.G.); 3 Beijing Sports Nutrition Engineering Research Center, Beijing Sport University, Xinxi Road No. 48, Beijing 100084, China; 4 Key Laboratory for Performance Training and Recovery of General Administration of Sport, Beijing Sport University, Xinxi Road No. 48, Beijing 100084, China; 5 Department of Health, Sport, and Hunman Physiology, University of Iowa, 101 Jessup Hall, Iowa City, IA 52240, USA; hongyinglu@bsu.edu.cn

**Keywords:** anterior cruciate ligament reconstruction, rehabilitation, strength, function

## Abstract

**Background/Objective:** Blood flow restriction (BFR) training has emerged as a promising adjunct to rehabilitation following anterior cruciate ligament reconstruction (ACLR), but its effectiveness across postoperative outcomes remains uncertain. To systematically evaluate and quantify the effects of BFR training on rehabilitation outcomes after ACLR. **Methods:** A systematic review and meta-analysis were conducted according to PRISMA guidelines. Ten electronic databases were searched from inception to December 2025. Eligible studies included randomized and non-randomized clinical trials investigating BFR after ACLR. Risk of bias was assessed using RoB 2 and ROBINS-I. Pooled standardized mean differences (SMDs) with 95% confidence intervals (CIs) were calculated using random-effects models, and certainty of evidence was evaluated using GRADE. **Results:** Seventeen studies involving 643 participants were included, of which 12 contributed to the meta-analysis. Most studies combined low-load resistance training with BFR at 40–80% limb occlusion pressure (LOP), initiated within 0–12 weeks after surgery and continued for 2–16 weeks. Compared with conventional rehabilitation, BFR significantly improved quadriceps strength (*n* = 9; SMD = 0.77, 95% CI 0.42–1.13; *p* < 0.0001; moderate-certainty evidence) and functional recovery (*n* = 6; SMD = 1.47, 95% CI 0.08–2.87; *p* = 0.04; I^2^ = 91%; low-certainty evidence). Larger effects were observed at occlusion pressures >80% LOP. No serious BFR-related adverse events were reported. No significant effects were found for balance (*n* = 5; SMD = 0.22, 95% CI −0.96 to 1.40; *p* = 0.72) or pain (*n* = 5; SMD = 0.82, 95% CI −0.28 to 1.92; *p* = 0.14), both supported by very low-certainty evidence. **Conclusions:** Moderate-certainty evidence supports BFR training for improving quadriceps strength after ACLR. Evidence for functional recovery is limited by substantial heterogeneity, while effects on pain, postural balance, and muscle morphology remain inconclusive. Findings regarding optimal occlusion pressure should be considered exploratory pending confirmation in future trials.

## 1. Introduction

One of the major challenges in early anterior cruciate ligament reconstruction (ACLR) rehabilitation is that high-load resistance training, typically required to stimulate muscle strength gains and promote quadriceps hypertrophy, is often contraindicated or poorly tolerated during the initial recovery phase [1,2]. This presents a clinical dilemma: while conventional low-intensity rehabilitation exercises are generally safe, they may be insufficient to prevent muscle atrophy and restore strength. Consequently, patients may experience prolonged muscle weakness and functional impairments that can persist for months or even years after surgery [3,4]. Thus, there is a clear need for rehabilitation strategies that can safely enhance muscular strength and function without imposing excessive mechanical stress on healing tissues. Blood flow restriction (BFR) training, when combined with low-load resistance exercise, has emerged as a promising therapeutic modality to address this gap [5]. This technique involves the application of a pneumatic cuff or tourniquet to the proximal portion of a limb in order to partially restrict arterial inflow and venous outflow during exercise. When performed with low-load resistance exercise (typically 20–30% of one-repetition maximum), BFR training has been shown to induce physiological adaptations comparable to those achieved with high-load resistance training [6,7,8]. In patients recovering from knee surgery, BFR may represent an effective strategy to attenuate disuse atrophy, accelerate strength recovery, and improve functional outcomes during the early phases of rehabilitation [9,10,11,12]. These characteristics make BFR particularly advantageous during early rehabilitation, when high mechanical loading may be contraindicated due to concerns related to joint integrity, graft protection, or soft tissue healing constraints [2,10,12].

The underlying mechanisms of BFR include the induction of a hypoxic and metabolite-rich intramuscular environment, which stimulates anabolic signaling pathways such as the mechanistic target of rapamycin (mTOR), promotes cellular swelling, enhances endocrine responses, and increases recruitment of fast-twitch muscle fibers [6,7,8,13,14,15,16,17,18]. These adaptations facilitate muscle hypertrophy and strength gains while minimizing joint stress, making BFR particularly suitable for post-operative populations [1,19,20,21]. Despite increasing interest in BFR training, the current evidence regarding its effectiveness in ACLR rehabilitation remains inconsistent, particularly with respect to functional performance and strength-related outcomes [1,21,22]. Therefore, a comprehensive synthesis of the available evidence is warranted.

Across recent systematic reviews, blood flow restriction (BFR) training following anterior cruciate ligament reconstruction (ACLR) or related knee surgeries has been reported to attenuate quadriceps atrophy and improve muscle strength. However, its effects on broader functional outcomes remain inconsistent. While some meta-analyses have demonstrated improvements in muscle strength and patient-reported knee function, others have reported no clear or consistent benefits for outcomes such as pain, range of motion, muscle morphology, or functional performance [21,22]. These discrepancies likely reflect differences in surgical populations, rehabilitation protocols, BFR application parameters, comparator interventions, outcome definitions, and follow-up durations, as well as the inclusion of relatively small and methodologically heterogeneous evidence bases in earlier reviews. Taken together, these limitations highlight the need for an updated and methodologically rigorous synthesis of the evidence.

This study aimed to systematically review and synthesize the evidence regarding the effectiveness of BFR training on rehabilitation outcomes in patients recovering from ACLR. We hypothesize that BFR training leads to significantly greater improvements in rehabilitation outcomes, particularly strength and function, compared to conventional rehabilitation, as indicated by pooled standardized mean differences (SMD) exceeding zero and outperforming control groups. Additionally, we hypothesized that the effectiveness of BFR varies according to occlusion pressure, timing of initiation following ACLR, and the duration of the intervention.

## 2. Materials and Methods

### 2.1. Study Design and Protocol Registration

This study was conducted in accordance with the Preferred Reporting Items for Systematic Reviews and Meta-Analyses (PRISMA) guidelines [23]. The methodology was structured using the PICO framework (Population, Intervention, Comparator, Outcome) to ensure systematic identification and synthesis of clinical studies evaluating the effectiveness of blood flow restriction (BFR) training in post-ACLR rehabilitation.

The review protocol was prospectively registered in PROSPERO (Registration No. CRD420251041431; registered on 28 April 2025). The protocol is available at: https://www.crd.york.ac.uk/PROSPERO/view/CRD420251041431 (accessed on 30 December 2025).

### 2.2. Data Sources and Search Strategy

A comprehensive literature search was conducted in the following electronic databases from inception to December 2025: PubMed/MEDLINE, Embase, Cochrane Central Register of Controlled Trials (CENTRAL), Scopus, Web of Science, EBSCOhost, ScienceDirect, SpringerLink, SAGE Journals, and Taylor & Francis Online. The search strategy combined controlled vocabulary terms (when available) and free-text keywords related to blood flow restriction (BFR) and anterior cruciate ligament reconstruction (ACLR). Boolean operators (AND, OR) were used to combine search terms, and database-specific syntax was adapted according to the indexing requirements of each database. The representative PubMed search strategy was as follows: (“blood flow restriction” OR BFR OR “vascular occlusion”) AND (“anterior cruciate ligament reconstruction” OR “ACL reconstruction” OR ACLR). The complete search strategies for all databases are provided in Supplementary Table S1.

### 2.3. Eligibility Criteria

Eligibility criteria were defined according to the PICO framework. Studies were included if they met the following criteria:

**Population** (P): Adults aged 18–60 years who underwent anterior cruciate ligament reconstruction (ACLR).

**Intervention** (I): Blood flow restriction (BFR) training implemented during postoperative rehabilitation within 6 months after ACLR and administered for a minimum duration of 2 weeks.

**Comparator** (C): Sham BFR, conventional rehabilitation or exercise, active control interventions, or no intervention.

**Outcomes** (O): Reporting at least one clinical outcome, including muscle strength, physical function, pain, range of motion, muscle morphology, or fatigue.

Study design: Randomized controlled trials, quasi-randomized trials, or retrospective comparative studies.

Studies were excluded if they met any of the following criteria:

1. Non-human studies (animal or in vitro studies).
2. Studies not involving participants who underwent ACLR.
3. BFR applied exclusively preoperatively or outside the postoperative rehabilitation period. Absence of a comparator group.
4. Non-original publications, including reviews, study protocols, editorials, letters, or case reports.
5. Insufficient data for extraction or analysis.
6. Duplicate publications or studies reporting overlapping datasets.

### 2.4. Outcomes

Primary outcomes were muscle strength, pain, range of motion (ROM), physical function, and muscle morphology.

**Muscle strength** was assessed as maximal voluntary force production using isokinetic or isometric dynamometry.

**Pain** was assessed as self-reported knee pain at rest or during activity, typically measured using a visual analogue scale (VAS) or numeric rating scale (NRS).

**Range of motion** (ROM) was assessed as active or passive knee flexion and/or extension using goniometric or equivalent clinical measurement tools.

**Physical function** was assessed using validated patient-reported outcome measures, including the International Knee Documentation Committee Subjective Knee Evaluation Form (IKDC), Knee Injury and Osteoarthritis Outcome Score (KOOS), and Lysholm Knee Score.

**Muscle morphology** was assessed as structural characteristics of the thigh musculature, such as muscle thickness or cross-sectional area, measured using ultrasound or magnetic resonance imaging (MRI).

**Fatigue resistance** was assessed as the decline in performance during repeated muscle contractions or exercise tasks.

**Adverse events** were defined as any intervention-related or study-reported negative health events occurring during the intervention period.

### 2.5. Study Selection and Screening Process

**Figure 1:**
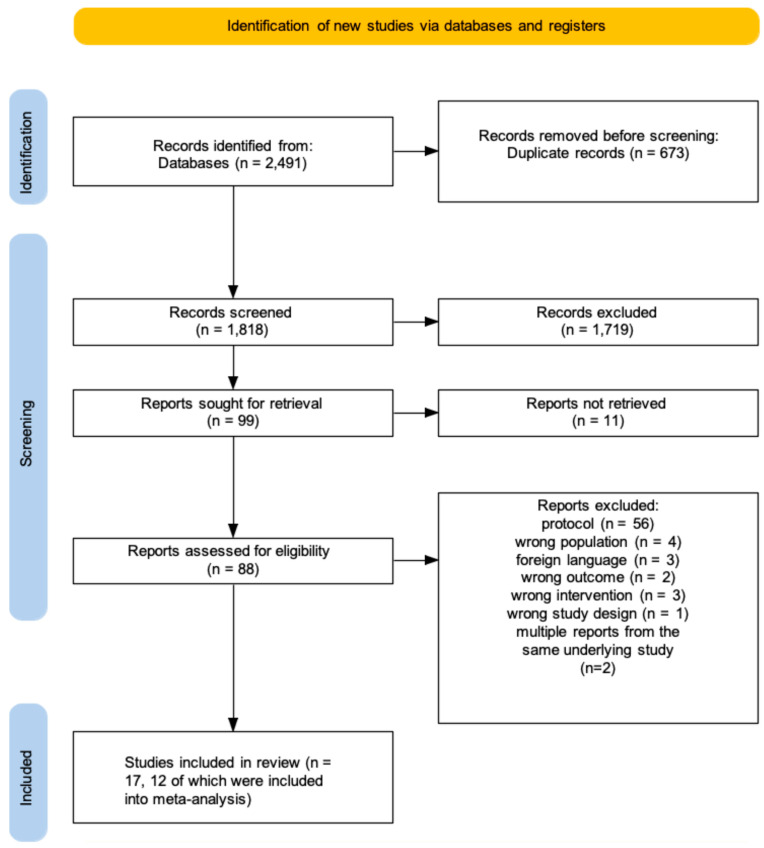
PRISMA flow diagram of study selection process.

Study selection was performed using Rayyan software (version 1.6.0) and followed a two-stage screening process. First, titles and abstracts were independently screened by NX, RG, and TW. Full-text eligibility assessment was subsequently conducted by the same reviewers. Disagreements were resolved through discussion and, when necessary, adjudication by VD. The study selection process is presented in the PRISMA flow [23] diagram (Figure 1).

### 2.6. Quality Assessment and Risk of Bias

Risk of bias was assessed according to study design. Randomized controlled trials were evaluated using the Cochrane Risk of Bias 2 (RoB 2) tool [24], while non-randomized studies were assessed using ROBINS-I [25].

Certainty of evidence was evaluated at the outcome level using the GRADE approach [26] and classified as high, moderate, low, or very low. Assessments were performed independently by NX and WT, with discrepancies resolved by consensus or consultation with VD.

### 2.7. Data Extraction

Data were independently extracted by NX and TW using a standardized data extraction form, with all entries subsequently verified by RG and VD. Extracted information included study characteristics (author, publication year, and study design), participant characteristics and sample size, intervention and comparator details (including blood flow restriction parameters), outcome measures and assessment time points, and quantitative outcome data, including means, standard deviations (SDs), and group sample sizes.

When outcome data were incomplete, values were extracted from figures or tables whenever possible. SDs were calculated from reported standard errors or confidence intervals, and medians with ranges were converted to means and SDs using validated statistical methods.

For studies reporting multiple measures within the same outcome domain, validated and widely used instruments were prioritized. Both composite and individual outcome measures were extracted when available. To avoid unit-of-analysis errors, multiple intervention arms were treated as separate comparisons, and only one outcome measure per domain was included in each meta-analysis. When multiple follow-up assessments were reported, post-intervention outcomes were prioritized according to a predefined hierarchy.

### 2.8. Data Synthesis

Qualitative and quantitative synthesis was based on predefined eligibility criteria. Study characteristics were tabulated and compared across outcome domains. Effect sizes were expressed as standardized mean differences (SMD). Evidence tables summarized study design, participant characteristics, methodological quality, and outcomes. All processes were performed by NX and TW and independently verified by RG, YH, and VD.

### 2.9. Statistical Analysis

Random-effects meta-analyses, subgroup analyses, and sensitivity analyses were performed using Review Manager (RevMan) version 5.3 and Stata Statistical Software version 15. Where appropriate, mean differences (MD) were used. Missing MD or SD values were imputed from confidence intervals or ranges using standard formulas. Standardized mean differences (SMD) with 95% confidence intervals were calculated. Individual study results were presented in tabular form, and pooled estimates were visually displayed using forest plots. Heterogeneity was assessed using the I^2^ statistic. Publication bias was not evaluated because the number of studies included in each outcome analysis was limited, and subgroup-specific analyses contained fewer than 10 studies. Subgroup analyses were conducted by outcome domain (e.g., strength, function, fatigue, morphology, endurance, bone mass) and by study quality level. A meta-regression was performed to explore the impact of sample size on effect sizes.

## 3. Results

### 3.1. Characteristics of the Included Studies

Seventeen studies published between 2000 and 2025 met the eligibility criteria and were included in the systematic review, of which 12 contributed to the meta-analysis. Study characteristics are summarized in Table 1. A total of 24 intervention comparisons were available for quantitative synthesis. Participants had a weighted mean age of 28.8 ± 7.6 years, and interventions were initiated at a mean of 3.35 weeks post-ACLR. Most studies evaluated blood flow restriction (BFR) combined with low-load resistance exercise during the early postoperative phase. Intervention duration ranged from 2 to 16 weeks, and comparators included conventional rehabilitation, low- and high-load resistance training, sham BFR, and other exercise-based interventions. Outcomes assessed included muscle strength, physical function, pain, range of motion, muscle morphology, balance, bone mass, and return to sport. Considerable clinical and methodological heterogeneity was observed across rehabilitation protocols, BFR parameters, outcome measures, and follow-up durations.

### 3.2. Risk of Bias Assessment

**Figure 2:**
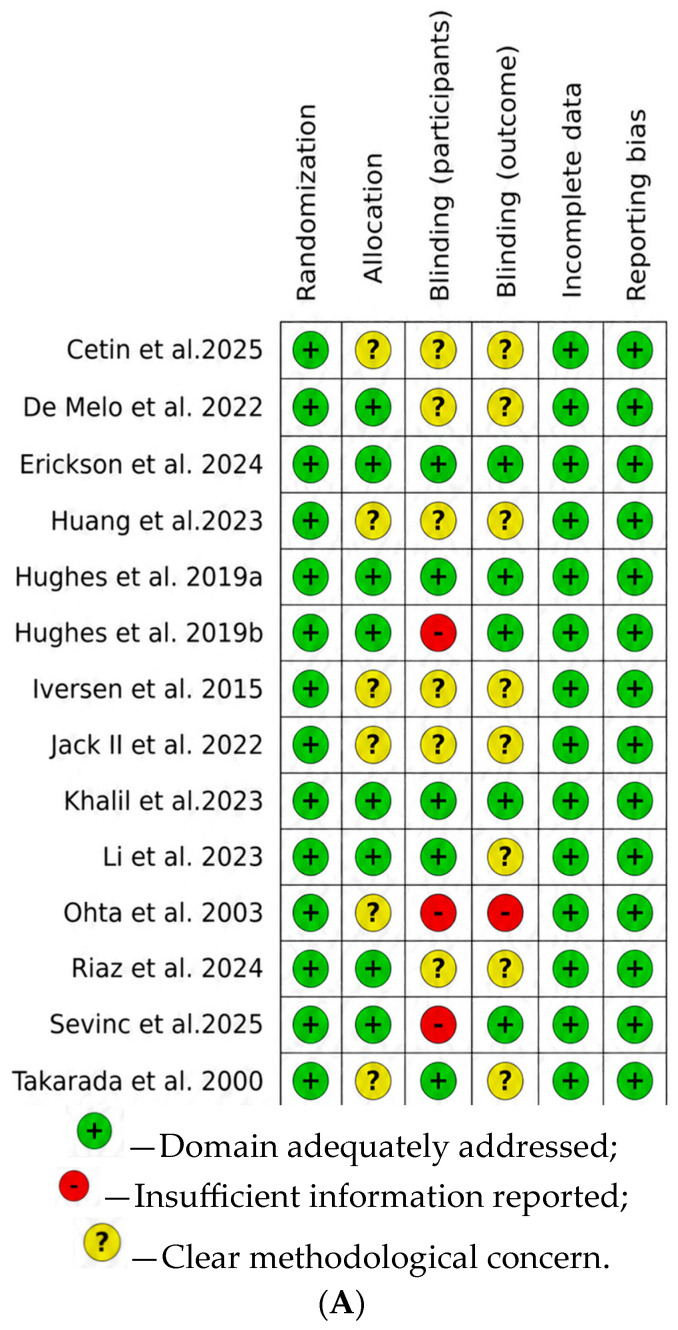

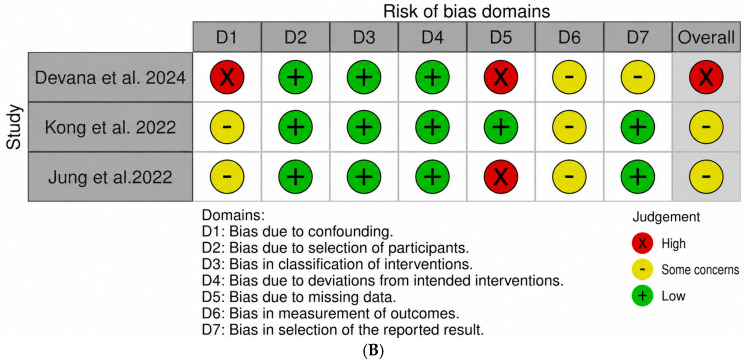
Risk of bias assessment using (**A**) RoB 2 (N = 14) [2,10,27,28,29,30,31,32,34,36,37,38,39,41] and (**B**) ROBINS-I (N = 3) [33,35,40].

Risk-of-bias assessments are presented in Figure 2. Among randomized controlled trials, most domains were judged as low risk of bias, although allocation concealment and blinding procedures were frequently insufficiently reported. The three non-randomized studies were judged to have moderate-to-serious overall risk of bias, primarily due to confounding and outcome measurement concerns.

### 3.3. Muscle Strength and Morphology

**Figure 3:**
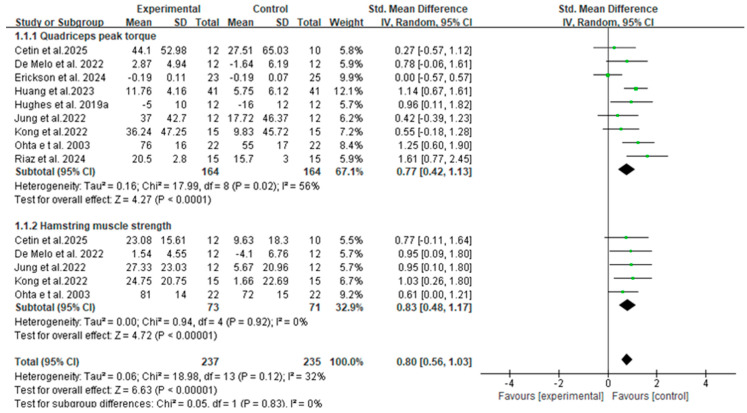
Overall Effect on Muscle Strength [2,10,27,28,29,30,33,35,36].

Nine randomized trials (*n* = 328) were included in the analysis of quadriceps muscle strength. Compared with control interventions, blood flow restriction (BFR) training produced a significant improvement in strength (SMD = 0.77, 95% CI 0.42–1.12; I^2^ = 56%; *p* < 0.0001), indicating moderate-certainty evidence in favour of BFR (Figure 3; Table S2).

Exploratory subgroup analyses examined the timing of BFR initiation, intervention duration, and occlusion pressure. Larger effect estimates were observed when BFR was initiated within 2 weeks postoperatively (SMD = 1.03, 95% CI 0.67–1.39; I^2^ = 7%; *p* < 0.00001) and for interventions lasting 4–8 weeks (SMD = 1.10, 95% CI 0.69–1.51; I^2^ = 0%; *p* < 0.00001) or longer than 8 weeks (SMD = 0.74, 95% CI 0.15–1.33; I^2^ = 71%; *p* = 0.01) (Supplementary Figures S1 and S2). No significant effects were observed for later initiation (2–8 weeks postoperatively) or for interventions lasting less than 4 weeks.

Similarly, studies using occlusion pressures greater than 80% of limb occlusion pressure showed larger effect estimates (SMD = 1.00, 95% CI 0.57–1.42; Supplementary Figure S3). However, the test for subgroup differences was not statistically significant, indicating insufficient evidence that treatment effects vary by occlusion pressure. Given that these analyses were exploratory, based on a limited number of studies, and involved multiple comparisons, the findings should be interpreted with caution. They may inform future research but do not provide definitive evidence that intervention timing, duration, or occlusion pressure modifies the effectiveness of BFR.

No overall significant effect of BFR training was observed for combined muscle morphology outcomes following ACL reconstruction. Although muscle thickness showed a statistically significant improvement, no significant differences were found for cross-sectional area or thigh circumference. Substantial between-study heterogeneity limits the certainty and generalisability of the pooled estimates (Supplementary Figure S4).

### 3.4. Lower Limbs Function

**Figure 4:**
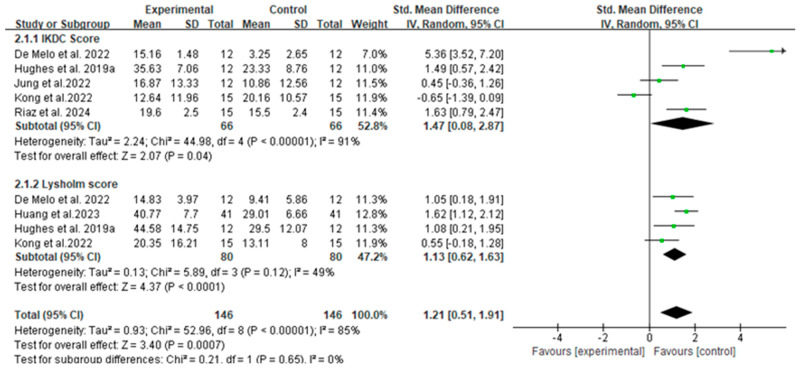
Overall Effect on Lower Limb Function [2,28,30,33,35,36].

A total of six randomized trials (*n* = 214) showed a statistically significant pooled effect of BFR training on lower limb function (IKDC score: SMD = 1.47, 95% CI 0.08–2.87; *p* = 0.04). However, this estimate should be interpreted with caution due to very high heterogeneity (I^2^ = 91%) and low certainty of evidence (Table S2; Figure 4). The wide confidence interval, spanning from a minimal to a large effect, reflects substantial inconsistency across studies and limits the interpretability of the overall pooled estimate.

Prespecified subgroup analyses demonstrated reduced heterogeneity and larger effects when the intervention duration was 4–8 weeks. In this subgroup, BFR significantly improved IKDC (SMD = 1.94, 95% CI 1.14–2.35; I^2^ = 0%; *p* < 0.00001) and Lysholm scores (SMD = 1.56, 95% CI 1.16–1.96; I^2^ = 39%; *p* < 0.00001) (Supplementary Figures S5 and S6), suggesting a more consistent signal than the overall pooled analysis. High-pressure BFR (>80% LOP) was also associated with greater functional improvement (SMD = 2.61, 95% CI 0.87–4.34; I^2^ = 86%; *p* = 0.003), whereas lower-pressure protocols showed no significant effect, with a statistically significant subgroup difference (*p* = 0.003) (Supplementary Figure S7). Nevertheless, substantial heterogeneity persisted within the >80% LOP subgroup, and each subgroup contained only a small number of studies. Therefore, these findings should be considered exploratory and insufficient to define definitive treatment thresholds.

An additional limitation is outcome heterogeneity across studies: functional recovery was assessed using IKDC (*n* = 6), Lysholm (*n* = 5), LEFS (*n* = 1), and KOOS (*n* = 1). Although standardized mean differences allow statistical pooling, they do not resolve differences in construct definition, scaling properties, or responsiveness across instruments. Consequently, pooling these measures as a single “functional outcome” should be interpreted as an approximation rather than a homogeneous construct, and may overstate the conceptual consistency of the evidence base.

### 3.5. Pain

**Figure 5:**
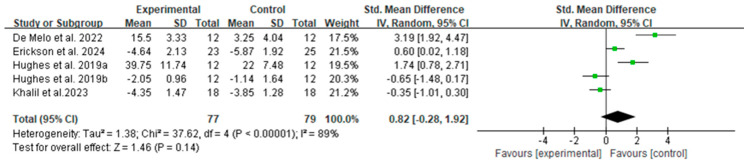
Overall Effect on Pain [2,28,29,31,34].

A total of five randomized trials (*n* = 156) evaluated the effects of BFR training on pain and symptoms. The pooled analysis showed a non-significant effect with substantial heterogeneity (SMD = 0.82, 95% CI −0.28 to 1.92; *p* = 0.14; I^2^ = 89%; very low certainty of evidence) (Figure 5; Table S2). The high degree of heterogeneity indicates marked inconsistency in both the direction and magnitude of effects across studies, with individual trials reporting effects ranging from potential harm to substantial benefit. Consequently, the pooled estimate does not represent a stable or generalizable effect and should be interpreted cautiously.

The certainty of evidence was rated as very low due to serious risk of bias, substantial inconsistency, and imprecision. Prespecified subgroup analyses based on intervention duration (4–8 weeks vs. >8 weeks) yielded non-significant and inconsistent effects and did not meaningfully reduce heterogeneity (Supplementary Figure S8). At the individual study level, some trials reported clinically relevant reductions in pain during or immediately after the intervention, whereas others found no additional benefit compared with conventional rehabilitation or reported increased exercise-related discomfort.

This variability likely reflects differences in pain constructs assessed (joint pain versus muscle discomfort), measurement instruments (e.g., VAS, NRS, and Borg-type scales), comparator intensity, and timing of outcome assessment. Taken together, the available evidence does not allow reliable conclusions regarding the effectiveness of BFR training for pain modulation after ACL reconstruction.

### 3.6. Balance

BFR training did not demonstrate significant effects on balance outcomes in the pooled analysis (Figure S9). Subgroup analyses based on intervention duration and BFR pressure yielded consistent null findings (Supplementary Figures S10 and S11). Substantial heterogeneity was observed, indicating variability across the included studies.

### 3.7. Intervention Pathway and Comparator

When stratified by intervention pathway and comparator, the effectiveness of BFR appeared to depend on the rehabilitation context. In protocols where BFR was added to low-load resistance training, several studies reported favorable outcomes compared with low-load training alone or usual care [10,27,28,30,33,37,38]. However, when compared with high-load resistance training or sham-controlled interventions, the relative advantage of BFR was less consistent, with some studies suggesting comparable efficacy rather than clear superiority [2,29,31]. Early ischemia-only protocols (without structured resistance progression) showed inconsistent anti-atrophy effects [39]. Alternative strategies, such as contralateral cross-education approaches, demonstrated limited or inconsistent effects, indicating that structured resistance training may be a key component for optimizing BFR efficacy [41].

### 3.8. Safety and Clinical Interpretation

Adverse event reporting across the 17 included studies was sparse and methodologically inconsistent, limiting robust conclusions regarding the safety profile of BFR in post-ACLR rehabilitation. Only one study [30] prespecified complication rates as an outcome and reported a lower postoperative complication rate in the BFR group (4.9% vs. 24.4%); however, complication types and ascertainment procedures were insufficiently detailed, limiting interpretability. Hughes et al. [31] systematically evaluated symptom responses during exercise and reported that BFR at 80% limb occlusion pressure (LOP) increased local muscle discomfort compared with high-load resistance training, while simultaneously reducing knee joint pain. This dissociation between local exertional discomfort and joint pain tolerance is clinically relevant but is based on a single study.

Two additional studies reported KOOS pain and effusion sub-scores as secondary outcomes [2,28], providing indirect and limited evidence of symptom tolerability. The remaining studies either did not report adverse events or stated only that no serious adverse events occurred, without specifying monitoring procedures, definitions, or surveillance methods. Consequently, systematic assessment of adverse events was not consistently undertaken across trials.

Local exercise-related discomfort was occasionally reported [10], whereas vascular safety outcomes were rarely systematically assessed. Overall, the available evidence suggests that BFR is generally well tolerated in post-ACLR populations when applied at moderate occlusion pressures (60–80% LOP) in combination with supervised low-load resistance training, consistent with broader safety findings in clinical populations. However, this interpretation is constrained by selective sampling, as most studies excluded individuals with cardiovascular disease, thromboembolic history, or peripheral vascular pathology.

Therefore, the current safety evidence does not extend to higher-risk populations, and careful screening and monitoring remain essential prior to clinical implementation. These limitations in adverse event reporting and participant selection likely contribute to between-study heterogeneity and should be considered when interpreting the overall evidence base for BFR in ACLR rehabilitation.

## 4. Discussion

This systematic review and meta-analysis suggest that blood flow restriction (BFR) may be a useful adjunct to rehabilitation following anterior cruciate ligament reconstruction (ACLR), particularly for improving quadriceps strength and patient-reported knee function when combined with low-load resistance exercise. Leave-one-out analyses indicated that pooled effects for muscle strength and patient-reported function were not driven by any single study, supporting the robustness of these findings. Overall, results are consistent with previous ACLR-focused systematic reviews, which have reported favourable effects of BFR on strength and selected functional outcomes, although certainty has generally been limited by small sample sizes, heterogeneous protocols, and imprecision [21,22,42]. Similarly, He et al. [43] reported no significant differences between low-load BFR and high-load resistance training for pain, strength, or functional outcomes, supporting the potential role of BFR when high-load exercise is not feasible.

Subgroup analyses suggested larger effects when BFR was initiated early (within two postoperative weeks), applied for 4–8 weeks, or performed using higher occlusion pressures. These findings are biologically plausible given early postoperative neuromuscular inhibition and reduced tolerance to high-load exercise. However, subgroup analyses were exploratory, based on limited study numbers, and generally not supported by significant interaction tests. Therefore, they should be interpreted as hypothesis-generating and used primarily to inform future trial design rather than clinical decision-making.

Although several included studies reported improvements in strength, function, balance, and muscle morphology, the certainty of evidence was low to very low according to GRADE, mainly due to risk of bias, inconsistency, and imprecision. Accordingly, further high-quality randomized controlled trials are required to strengthen confidence in these findings.

From a mechanistic perspective, quadriceps weakness following ACLR is a major functional limitation that may persist long-term and is not fully explained by muscle atrophy alone [44]. Arthrogenic muscle inhibition and altered neuromuscular activation are key contributors to impaired recovery [45]. In this context, BFR may provide a physiological stimulus that allows low-load exercise to induce meaningful adaptations through metabolic stress and hypoxia-driven signaling [46,47]. Proposed mechanisms include increased motor unit recruitment (particularly type II fibers), activation of anabolic pathways such as mTOR, transient increases in growth hormone and insulin-like growth factor 1 (IGF-1), and reductions in myostatin expression [48]. Evidence also suggests increased myogenic stem cell activity and myonuclear accretion, providing a biological basis for strength improvements during early rehabilitation [49].

A notable finding was the discrepancy between strength and muscle morphology outcomes. While quadriceps strength improved, pooled morphological outcomes were inconsistent and largely non-significant. This may reflect the fact that early strength gains are primarily driven by neural adaptations, whereas hypertrophy requires longer intervention duration. In addition, morphological outcomes were highly heterogeneous due to differences in imaging modality, timing, graft type, and anatomical measurement sites [44,50]. Therefore, the absence of significant pooled morphological effects should be interpreted cautiously.

Functional outcomes showed substantial heterogeneity. Nevertheless, interventions lasting 4–8 weeks and using higher occlusion pressures were more consistently associated with improved outcomes. Patient-reported outcome measures (e.g., IKDC, Lysholm) are multidimensional and reflect symptoms, confidence, and perceived function, meaning observed improvements may reflect broader rehabilitation effects rather than isolated physiological adaptations. This may also explain inconsistent findings across previous meta-analyses [21,22].

Pressure-related subgroup analysis suggested that higher occlusion pressures (>80% LOP) may be associated with greater improvements in strength and function, with a significant subgroup difference observed for functional outcomes. This aligns with Li et al. [38], who reported superior outcomes at 80% versus 40% LOP. However, higher pressures are not universally superior, as cuff width, limb morphology, and device characteristics influence the physiological stimulus [19,51]. Adverse event reporting was inconsistent and largely passive. Although no serious events were reported at higher pressures, tolerability may decrease due to increased discomfort [31]. Overall, current evidence most cautiously supports 60–80% LOP, consistent with consensus recommendations [19,51], while the superiority of >80% LOP remains uncertain.

Between-study heterogeneity was also influenced by variation in graft type and rehabilitation timing. Different graft types may affect donor-site morbidity and neuromuscular recovery [52,53], but most studies did not report graft type, limiting subgroup analysis. Similarly, initiation timing ranged from days to weeks postoperatively, contributing to residual heterogeneity despite subgrouping.

Pain, balance, safety, and endocrine outcomes remain highly uncertain. Pain outcomes were highly heterogeneous due to differences in constructs and measurement timing. Safety data were limited by passive reporting and lack of standardized vascular monitoring. Given the elevated thromboembolic risk following ACLR [42,54,55], the true incidence of rare adverse events cannot be determined. Preliminary evidence suggests possible endocrine and endurance-related adaptations, but findings are inconsistent and based on small heterogeneous samples [56,57], and should be considered exploratory.

Outcome reporting across studies was inconsistent, and clinically important endpoints such as return-to-sport, reinjury rates, and neuromuscular activation were rarely reported [29,33,37,40]. In addition, heterogeneity in outcome definitions limits interpretation of standardized mean differences, which should therefore be considered broad indicators rather than precise effect estimates [19,58].

This review has several limitations. Considerable variation existed in BFR protocols, including occlusion pressure, exercise load, frequency, and duration. Outcome measures and follow-up periods were also inconsistent, contributing to statistical heterogeneity. Publication bias cannot be excluded, particularly where few studies contributed to outcomes. In addition, limited study numbers restricted subgroup and meta-regression analyses, reducing the ability to fully explore heterogeneity.

The available evidence suggests that BFR may be considered as an adjunct to low-load resistance exercise during early ACLR rehabilitation, particularly when high-load training is not feasible. However, the certainty of evidence ranged from low to moderate, and substantial heterogeneity was observed. Therefore, current findings do not support a standardized BFR protocol. Clinical application should be individualized according to rehabilitation stage, patient tolerance, occlusion pressure, cuff characteristics, and treatment goals.

Future research should focus on adequately powered randomized controlled trials using standardized BFR protocols and consistent outcome measures. Studies should include comprehensive adverse-event reporting, longer follow-up periods, and clinically meaningful endpoints such as return-to-sport and reinjury rates. Further work is also required to clarify optimal occlusion pressure, timing, and progression strategies, and to better understand the mechanisms underlying neuromuscular and morphological adaptations following BFR in ACLR rehabilitation.

## 5. Conclusions

BFR training may confer short-term benefits for quadriceps strength and selected functional outcomes following ACLR; however, the certainty of evidence is low to very low, and substantial heterogeneity limits confidence in pooled estimates. Accordingly, BFR should be considered a supportive adjunct during early rehabilitation rather than a definitive or universally effective intervention.

## Figures and Tables

**Table 1:** Characteristics of Included Studies.

Study	Synthesis Type	Sample Size	Age (Years)	Time Since ACLR	Intervention Protocol	Outcomes
Çetin et al., 2025 [27]	MA	LL: 10; LL + BFR: 12	33.2 ± 11.8; 28.6 ± 8.6	8 weeks	4 weeks; 3×/week; 20% 1RM; 80% LOP	Strength
De Melo et al., 2022 [28]	MA	HL: 12; LL + BFR: 12	39.6 ± 10.8; 41.1 ± 9.8	Post-discharge	12 weeks; 2×/week; 30% 1RM; 80% LOP	Strength; function; pain
Erickson et al., 2024 [29]	MA	HL + sham: 25; LL + BFR: 23	21.5 ± 5.3; 21.1 ± 6.3	2 months	4–5 months; 3×/week; 20% 1RM; 60% LOP	Strength; muscle morphology
Huang et al., 2023 [30]	MA	CR: 41; CR + BFR: 41	29.5 ± 4.2; 29.1 ± 3.9	<6 weeks	6 weeks; 5×/week; 30% 1RM; 75 mmHg	Strength; function; balance
Hughes et al., 2019a [2]	MA	HL: 12; LL + BFR: 12	29 ± 7	2 weeks	8 weeks; 2×/week; 30% 1RM; 80% LOP	Strength; function; ROM; pain
Hughes et al., 2019b [31]	MA	HL: 12; LL + BFR: 12	28.7 ± 7.2; 29.4 ± 6.8	2 weeks	8 weeks; 2×/week	Pain
Iversen et al., 2016 [32]	MA	CR: 12; CR + BFR: 12	29.8 ± 9.3; 24.9 ± 7.4	2 days	2 weeks; 2×/day; 130–180 mmHg	Muscle morphology
Jung et al., 2022 [33]	MA	CR: 12; CR + BFR: 12	27.8 ± 8.4; 30.8 ± 7.6	4 weeks	12 weeks; 3×/week; 40% LOP	Strength; function; balance
Khalil et al., 2023 [34]	MA	CR: 18; CR + BFR: 18	25.2 ± 4.8; 23.8 ± 3.9	1 week	12 weeks; 80% LOP	Pain
Kong et al., 2022 [35]	MA	CR: 15; CR + BFR: 15	27.5 ± 8.4; 29.1 ± 9.1	4 weeks	12 weeks; 3×/week; 40% LOP	Strength; function; balance
Ohta et al., 2003 [10]	MA	LL: 22; LL + BFR: 22	30 ± 9.7; 28 ± 9.7	2 weeks	16 weeks; ~20% 1RM; 180 mmHg	Strength; muscle morphology
Riaz et al., 2024 [36]	MA	PNF: 15; BFR: 15	35 ± 5.2	2 weeks	10 weeks	Strength; function
Jack et al., 2023 [37]	QO	CR: 15; CR + BFR: 17	24.8 ± 3.9; 25.3 ± 4.1	<1 week	12 weeks; 2×/week; 80% LOP	Strength; function; bone mass; Return to sport
Li et al., 2023 [38]	QO	CR: 6; BFR40%: 9; BFR80%: 8	~23	>8 weeks	8 weeks; 2×/week	Strength; morphology; function
Takarada et al., 2000 [39]	QO	C: 8; BFR: 8	~23	3 days	2 weeks; 2×/day	Muscle morphology
Devana et al., 2024 [40]	QO	CR: 33; BFR: 22	~19–20	10–14 days	~52 weeks	Return to sport
Sevinc et al., 2025 [41]	QO	CR: 11; BFR: 13	~25	4 weeks	8 weeks	Strength; morphology

Abbreviations: ACLR, anterior cruciate ligament reconstruction; BFR, blood flow restriction; C, control group; CR, conventional rehabilitation; HL, high-load resistance training; LL, low-load resistance training; PNF, proprioceptive neuromuscular facilitation; 1RM, one-repetition maximum; LOP, limb occlusion pressure; ROM, range of motion; MA, meta-analysis; QO, qualitative synthesis only.

## Data Availability

All data underlying this study are derived from publicly available journal articles.

## Supplementary Materials

The following supporting information can be downloaded at https://www.mdpi.com/article/10.3390/jcm15124706/s1, Figure S1. Subgroup analysis of muscle strength according to timing of BFR initiation after ACL reconstruction (ACLR); Figure S2. Subgroup Analysis of Muscle Strength According to Intervention Duration; Figure S3. Subgroup analysis of muscle strength according to blood flow restriction (BFR) pressure; Figure S4. Overall effect of muscle morphology outcomes; Figure S5. Subgroup Analysis of IKDC Score According to Intervention Duration; Figure S6. Subgroup Analysis of Lysholm Score According to Intervention Duration; Figure S7. Subgroup analysis of IKDC score according to blood flow restriction (BFR) pressure; Figure S8. Subgroup Analysis of Pain According to Intervention Duration; Figure S9. Overall Effect of Balance; Figure S10. Subgroup analysis of balance according to blood flow restriction (BFR) pressure; Figure S11. Subgroup Analysis of Balance According to Intervention Duration; Table S1. Search strategies used across electronic databases; Table S2. Certainty of Evidence Assessment Using the GRADE Approach; Table S3. PRISMA checklist; Table S4. PRISMA abstract checklist.

## Abbreviations

The following abbreviations are used in this manuscript:

1RM	One-Repetition Maximum
ACLR	Anterior Cruciate Ligament Reconstruction
BFR	Blood Flow Restriction
CI	Confidence Interval
GRADE	Grading of Recommendations Assessment, Development and Evaluation
IGF-1	Insulin-like Growth Factor 1
IKDC	International Knee Documentation Committee Subjective Knee Evaluation Form
KOOS	Knee Injury and Osteoarthritis Outcome Score
LOP	Limb Occlusion Pressure
MD	Mean Difference
MRI	Magnetic Resonance Imaging
mTOR	mechanistic Target of Rapamycin
NRS	Numeric Rating Scale
PICO	Population, Intervention, Comparator, Outcome
PRISMA	Preferred Reporting Items for Systematic Reviews and Meta-Analyses
RoB 2	Revised Cochrane Risk of Bias Tool for Randomized Trials
ROBINS-I	Risk of Bias in Non-randomized Studies of Interventions
ROM	Range of Motion
SD	Standard Deviation
SMD	Standardized Mean Difference
VAS	Visual Analogue Scale
