# Supplementary material for: The Effectiveness of Blood Flow Restriction Training on Rehabilitation After Anterior Cruciate Ligament Reconstruction: A Systematic Review and Meta-Analysis
Source: J Clin Med. 2026 Jun 17;15(12):4706. doi: 10.3390/jcm15124706 (PMC13302091; doi:10.3390/jcm15124706)
Supplement: Supplementary file 1 [file jcm-15-04706-s001.zip › jcm-4334673-supplementary.pdf]

Study or Subgroup	Experimental Mean	Experimental SD	Experimental Total	Control Mean	Control SD	Control Total	Weight	Std. Mean Difference IV, Random, 95% CI
**1.2.1 Acute Phase - Within 2 Weeks**								
De Melo et al. 2022	2.87	4.94	12	-1.64	6.19	12	9.6%	0.78 [-0.06, 1.61]
Hughes et al. 2019a	-5	10	12	-16	12	12	9.4%	0.96 [0.11, 1.82]
Kong et al.2022	36.24	47.25	15	9.83	45.72	15	11.0%	0.55 [-0.18, 1.28]
Ohta e t al. 2003	76	16	22	55	17	22	12.2%	1.25 [0.60, 1.90]
Riaz et al. 2024	20.5	2.8	15	15.7	3	15	9.6%	1.61 [0.77, 2.45]
**Subtotal (95% CI)**			**76**			**76**	**51.8%**	**1.03 [0.67, 1.39]**
Heterogeneity: Tau² = 0.01; Chi² = 4.28, df = 4 (P = 0.37); I² = 7%								
Test for overall effect: Z = 5.66 (P < 0.00001)								
**1.2.2 Subacute Phase - 2-8 Weeks**								
Cetin et al.2025	44.1	52.98	12	27.51	65.03	10	9.5%	0.27 [-0.57, 1.12]
Erickson et al. 2024	-0.19	0.11	23	-0.19	0.07	25	13.5%	0.00 [-0.57, 0.57]
Huang et al.2023	11.76	4.16	41	5.75	6.12	41	15.2%	1.14 [0.67, 1.61]
Jung et al.2022	37	42.7	12	17.72	46.37	12	9.9%	0.42 [-0.39, 1.23]
**Subtotal (95% CI)**			**88**			**88**	**48.2%**	**0.49 [-0.11, 1.08]**
Heterogeneity: Tau² = 0.25; Chi² = 10.15, df = 3 (P = 0.02); I² = 70%								
Test for overall effect: Z = 1.60 (P = 0.11)								
**Total (95% CI)**			**164**			**164**	**100.0%**	**0.77 [0.42, 1.13]**
Heterogeneity: Tau² = 0.16; Chi² = 17.99, df = 8 (P = 0.02); I² = 56%								
Test for overall effect: Z = 4.27 (P < 0.0001)								
Test for subgroup differences: Chi² = 2.34, df = 1 (P = 0.13), I² = 57.3%								

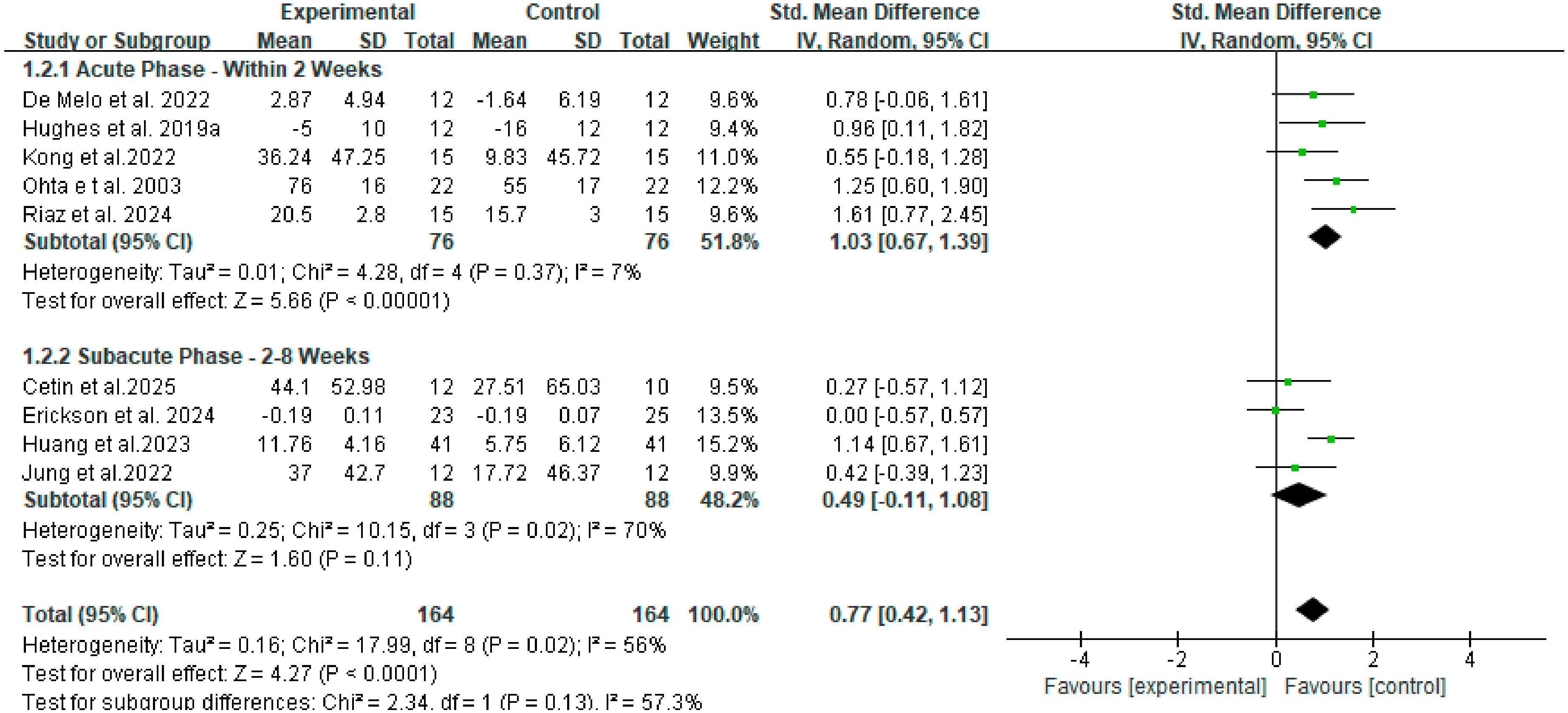

Figure S1. Subgroup analysis of muscle strength according to timing of BFR initiation after ACL reconstruction (ACLR)

Study or Subgroup	Experimental Mean	Experimental SD	Experimental Total	Control Mean	Control SD	Control Total	Weight	Std. Mean Difference IV, Random, 95% CI
**1.3.1 Short Term - Less Than 4 Weeks**								
Cetin et al.2025	44.1	52.98	12	27.51	65.03	10	9.5%	0.27 [-0.57, 1.12]
De Melo et al. 2022	2.87	4.94	12	-1.64	6.19	12	9.6%	0.78 [-0.06, 1.61]
**Subtotal (95% CI)**			**24**			**22**	**19.1%**	**0.53 [-0.07, 1.12]**
Heterogeneity: Tau² = 0.00; Chi² = 0.70, df = 1 (P = 0.40); I² = 0%								
Test for overall effect: Z = 1.74 (P = 0.08)								
**1.3.2 Moderate Term - 4-8 Weeks**								
Huang et al.2023	11.76	4.16	41	5.75	6.12	41	15.2%	1.14 [0.67, 1.61]
Hughes et al. 2019a	-5	10	12	-16	12	12	9.4%	0.96 [0.11, 1.82]
**Subtotal (95% CI)**			**53**			**53**	**24.6%**	**1.10 [0.69, 1.51]**
Heterogeneity: Tau² = 0.00; Chi² = 0.13, df = 1 (P = 0.72); I² = 0%								
Test for overall effect: Z = 5.24 (P < 0.00001)								
**1.3.3 Long Term - More Than 8 Weeks**								
Erickson et al. 2024	-0.19	0.11	23	-0.19	0.07	25	13.5%	0.00 [-0.57, 0.57]
Jung et al.2022	36.24	47.25	15	9.83	45.72	15	11.0%	0.55 [-0.18, 1.28]
Kong et al.2022	37	42.7	12	17.72	46.37	12	9.9%	0.42 [-0.39, 1.23]
Ohta e t al. 2003	76	16	22	55	17	22	12.2%	1.25 [0.60, 1.90]
Riaz et al. 2024	20.5	2.8	15	15.7	3	15	9.6%	1.61 [0.77, 2.45]
**Subtotal (95% CI)**			**87**			**89**	**56.2%**	**0.74 [0.15, 1.33]**
Heterogeneity: Tau² = 0.31; Chi² = 13.71, df = 4 (P = 0.008); I² = 71%								
Test for overall effect: Z = 2.47 (P = 0.01)								
**Total (95% CI)**			**164**			**164**	**100.0%**	**0.77 [0.42, 1.13]**
Heterogeneity: Tau² = 0.16; Chi² = 17.99, df = 8 (P = 0.02); I² = 56%								
Test for overall effect: Z = 4.27 (P < 0.0001)								
Test for subgroup differences: Chi² = 2.64, df = 2 (P = 0.27), I² = 24.3%								

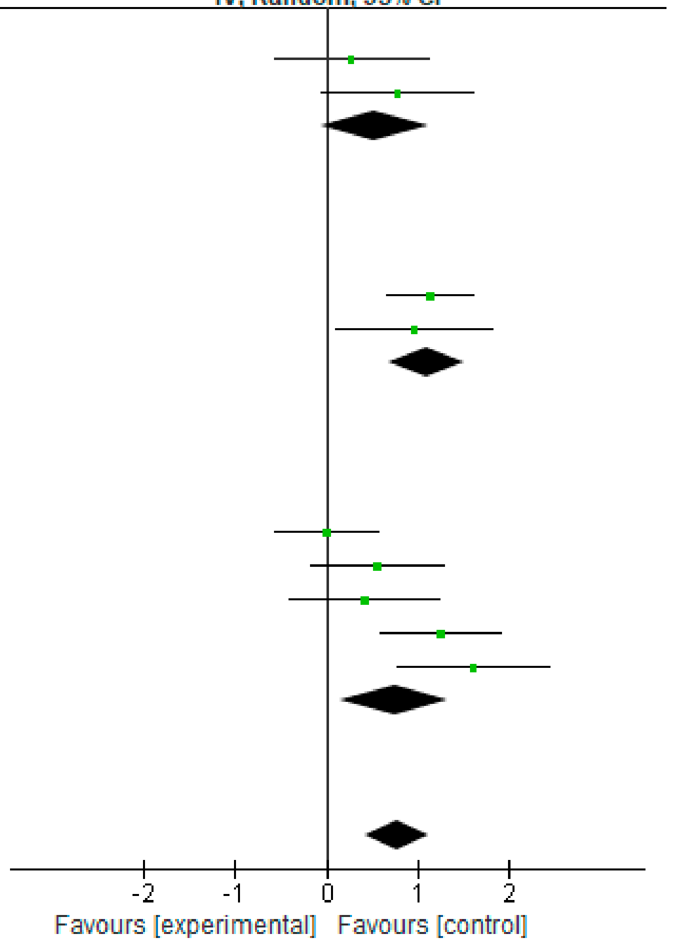

Figure S2. Subgroup analysis of muscle strength according to intervention duration

Study or Subgroup	Experimental Mean	Experimental SD	Experimental Total	Control Mean	Control SD	Control Total	Weight	Std. Mean Difference IV, Random, 95% CI
**1.4.1 Low Pressure - Below 60% LOP**								
Jung et al.2022	37	42.7	12	17.72	46.37	12	9.9%	0.42 [-0.39, 1.23]
Kong et al.2022	36.24	47.25	15	9.83	45.72	15	11.0%	0.55 [-0.18, 1.28]
**Subtotal (95% CI)**			**27**			**27**	**20.9%**	**0.49 [-0.05, 1.04]**
Heterogeneity: Tau² = 0.00; Chi² = 0.06, df = 1 (P = 0.81); I² = 0%								
Test for overall effect: Z = 1.78 (P = 0.08)								
**1.4.2 Moderate Pressure - 60%-80% LOP**								
Erickson et al. 2024	-0.19	0.11	23	-0.19	0.07	25	13.5%	0.00 [-0.57, 0.57]
Huang et al.2023	11.76	4.16	41	5.75	6.12	41	15.2%	1.14 [0.67, 1.61]
**Subtotal (95% CI)**			**64**			**66**	**28.7%**	**0.58 [-0.53, 1.70]**
Heterogeneity: Tau² = 0.58; Chi² = 9.21, df = 1 (P = 0.002); I² = 89%								
Test for overall effect: Z = 1.02 (P = 0.31)								
**1.4.3 High Pressure - Over 80% LOP**								
Cetin et al.2025	44.1	52.98	12	27.51	65.03	10	9.5%	0.27 [-0.57, 1.12]
De Melo et al. 2022	2.87	4.94	12	-1.64	6.19	12	9.6%	0.78 [-0.06, 1.61]
Hughes et al. 2019a	-5	10	12	-16	12	12	9.4%	0.96 [0.11, 1.82]
Ohta e t al. 2003	76	16	22	55	17	22	12.2%	1.25 [0.60, 1.90]
Riaz et al. 2024	20.5	2.8	15	15.7	3	15	9.6%	1.61 [0.77, 2.45]
**Subtotal (95% CI)**			**73**			**71**	**50.3%**	**1.00 [0.57, 1.42]**
Heterogeneity: Tau² = 0.07; Chi² = 5.73, df = 4 (P = 0.22); I² = 30%								
Test for overall effect: Z = 4.57 (P < 0.00001)								
**Total (95% CI)**			**164**			**164**	**100.0%**	**0.77 [0.42, 1.13]**
Heterogeneity: Tau² = 0.16; Chi² = 17.99, df = 8 (P = 0.02); I² = 56%								
Test for overall effect: Z = 4.27 (P < 0.0001)								
Test for subgroup differences: Chi² = 2.18, df = 2 (P = 0.34), I² = 8.3%								

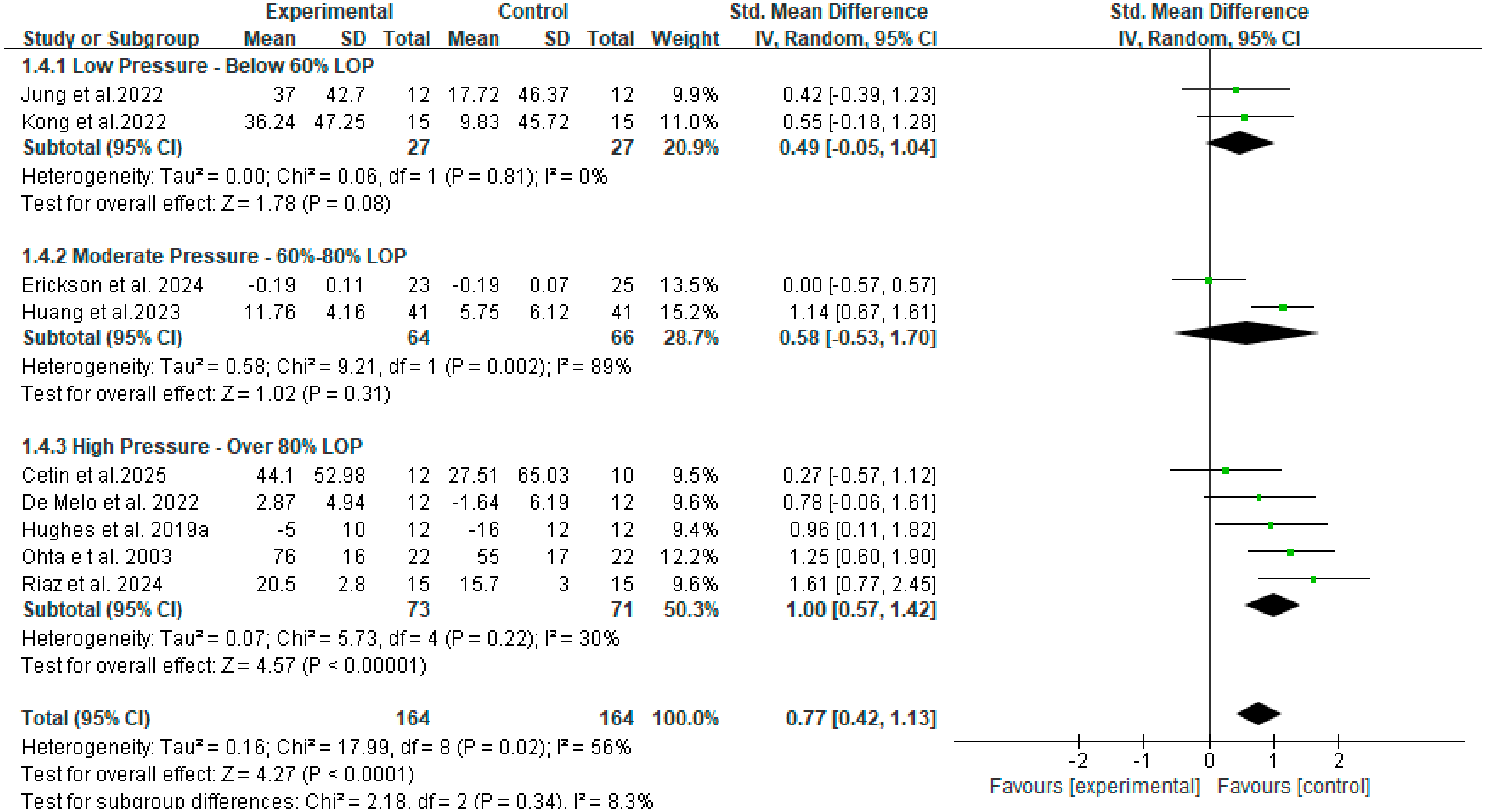

Figure S3. Subgroup analysis of muscle strength according to blood flow restriction (BFR) pressure

	Experimental			Control				Std. Mean Difference
Study or Subgroup	Mean	SD	Total	Mean	SD	Total	Weight	IV, Random, 95% CI
**3.1.1 Cross-Sectional Area**								
Erickson et al. 2024	102.7	203.79	23	61.42	172.66	25	14.7%	0.22 [-0.35, 0.78]
Iversen et al. 2015	-13.1	3.6	12	-11.5	3.3	12	13.3%	-0.45 [-1.26, 0.36]
Ohta e t al. 2003	101	11	22	92	12	22	14.5%	0.77 [0.15, 1.38]
**Subtotal (95% CI)**			**57**			**59**	**42.5%**	**0.22 [-0.41, 0.86]**
Heterogeneity: Tau² = 0.20; Chi² = 5.55, df = 2 (P = 0.06); I² = 64%								
Test for overall effect: Z = 0.69 (P = 0.49)								
**3.1.2 Thigh circumference**								
Huang et al.2023	-1.08	0.27	41	-0.64	0.22	41	15.0%	-1.77 [-2.28, -1.26]
Kong et al.2022	0.72	3.04	15	0.37	2.42	15	13.9%	0.12 [-0.59, 0.84]
**Subtotal (95% CI)**			**56**			**56**	**28.9%**	**-0.84 [-2.70, 1.02]**
Heterogeneity: Tau² = 1.69; Chi² = 17.71, df = 1 (P < 0.0001); I² = 94%								
Test for overall effect: Z = 0.89 (P = 0.37)								
**3.1.3 Muscle thickness**								
Huang et al.2023	-0.62	0.29	41	-0.42	0.24	41	15.4%	-0.74 [-1.19, -0.30]
Hughes et al. 2019a	0.1	0.04	12	0.12	0.06	12	13.3%	-0.38 [-1.19, 0.43]
**Subtotal (95% CI)**			**53**			**53**	**28.6%**	**-0.66 [-1.05, -0.27]**
Heterogeneity: Tau² = 0.00; Chi² = 0.60, df = 1 (P = 0.44); I² = 0%								
Test for overall effect: Z = 3.29 (P = 0.0010)								
**Total (95% CI)**			**166**			**168**	**100.0%**	**-0.33 [-1.00, 0.34]**
Heterogeneity: Tau² = 0.70; Chi² = 49.82, df = 6 (P < 0.00001); I² = 88%								
Test for overall effect: Z = 0.97 (P = 0.33)								
Test for subgroup differences: Chi² = 5.59, df = 2 (P = 0.06), I² = 64.2%								

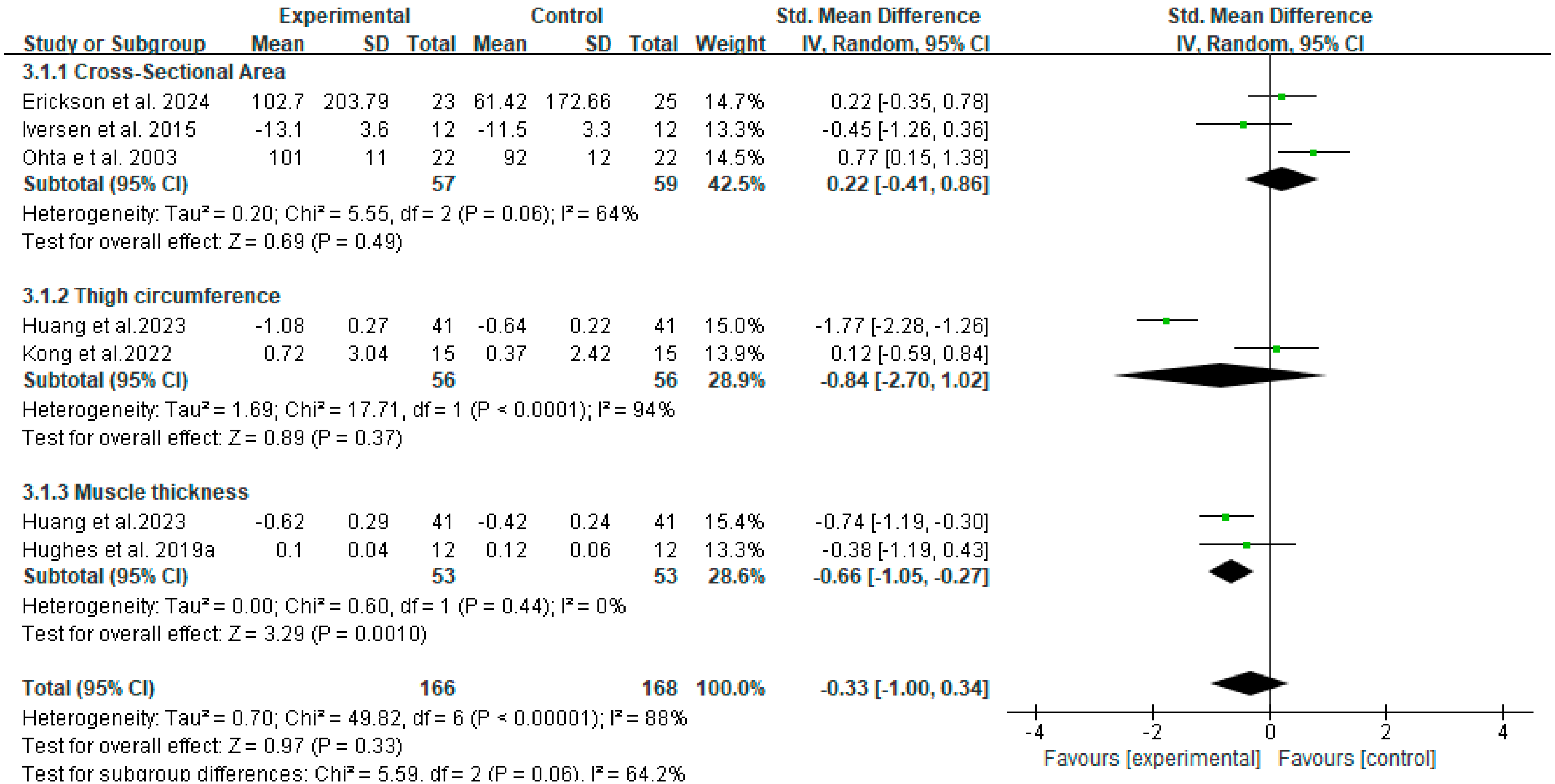

Figure S4. Overall effect of muscle morphology outcomes

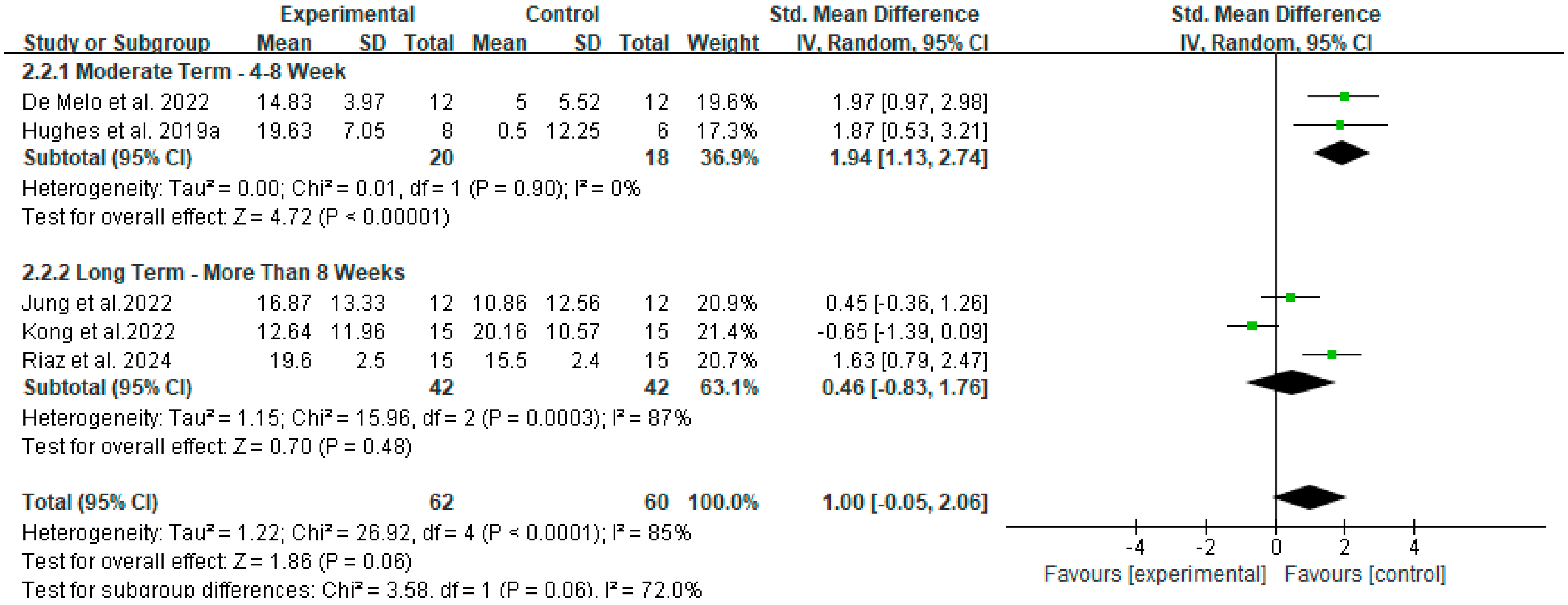

Figure S5. Subgroup analysis of IKDC score according to intervention duration

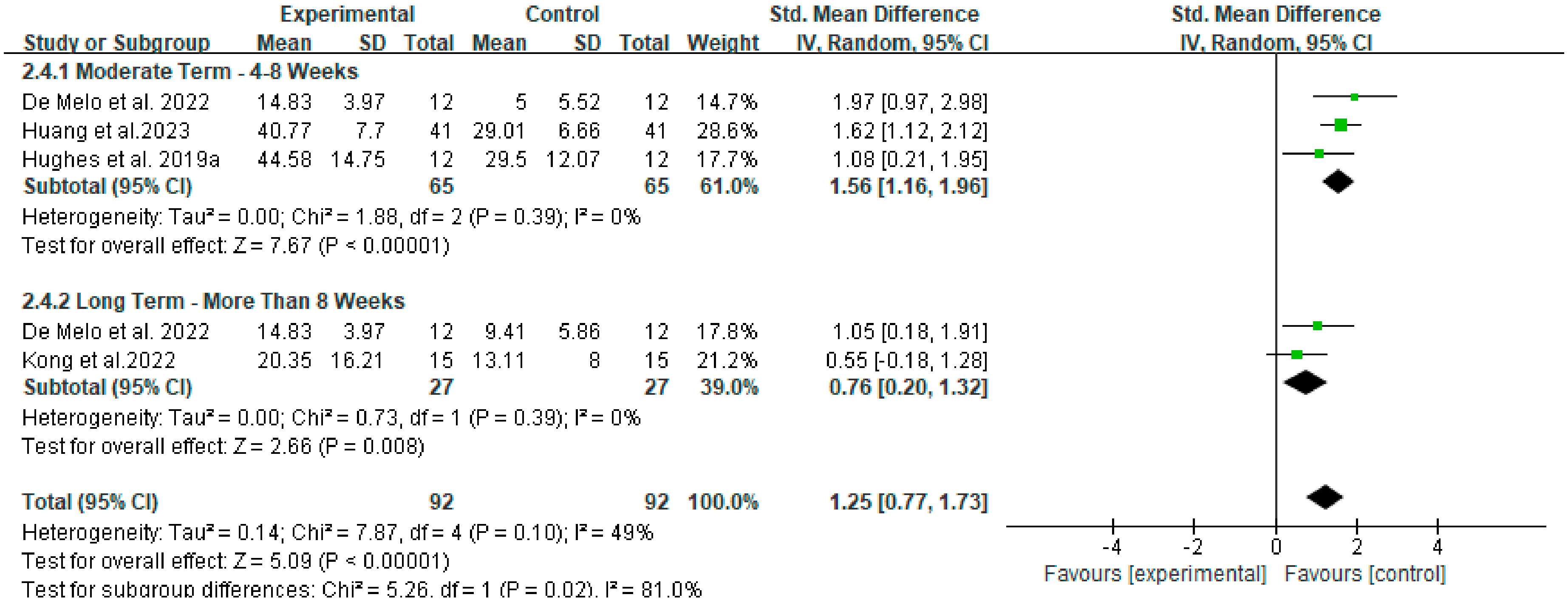

Figure S6. Subgroup analysis of Lysholm score according to intervention duration

Study or Subgroup	Experimental Mean	Experimental SD	Experimental Total	Control Mean	Control SD	Control Total	Weight	Std. Mean Difference IV, Random, 95% CI
**2.3.1 Low Pressure - Below 60% LOP**								
Jung et al.2022	16.87	13.33	12	10.86	12.56	12	21.0%	0.45 [-0.36, 1.26]
Kong et al.2022	12.64	11.96	15	20.16	10.57	15	21.3%	-0.65 [-1.39, 0.09]
**Subtotal (95% CI)**			**27**			**27**	**42.3%**	**-0.11 [-1.19, 0.96]**
Heterogeneity: Tau² = 0.44; Chi² = 3.84, df = 1 (P = 0.05); I² = 74%								
Test for overall effect: Z = 0.21 (P = 0.84)								
**2.3.3 High Pressure - Over 80% LOP**								
De Melo et al. 2022	15.16	1.48	12	3.25	2.65	12	16.2%	5.36 [3.52, 7.20]
Hughes et al. 2019a	35.63	7.06	12	23.33	8.76	12	20.6%	1.49 [0.57, 2.42]
Riaz et al. 2024	19.6	2.5	15	15.5	2.4	15	20.9%	1.63 [0.79, 2.47]
**Subtotal (95% CI)**			**39**			**39**	**57.7%**	**2.61 [0.87, 4.34]**
Heterogeneity: Tau² = 1.96; Chi² = 14.67, df = 2 (P = 0.0007); I² = 86%								
Test for overall effect: Z = 2.94 (P = 0.003)								
**Total (95% CI)**			**66**			**66**	**100.0%**	**1.47 [0.08, 2.87]**
Heterogeneity: Tau² = 2.24; Chi² = 44.98, df = 4 (P < 0.00001); I² = 91%								
Test for overall effect: Z = 2.07 (P = 0.04)								
Test for subgroup differences: Chi² = 6.83, df = 1 (P = 0.009), I² = 85.4%								

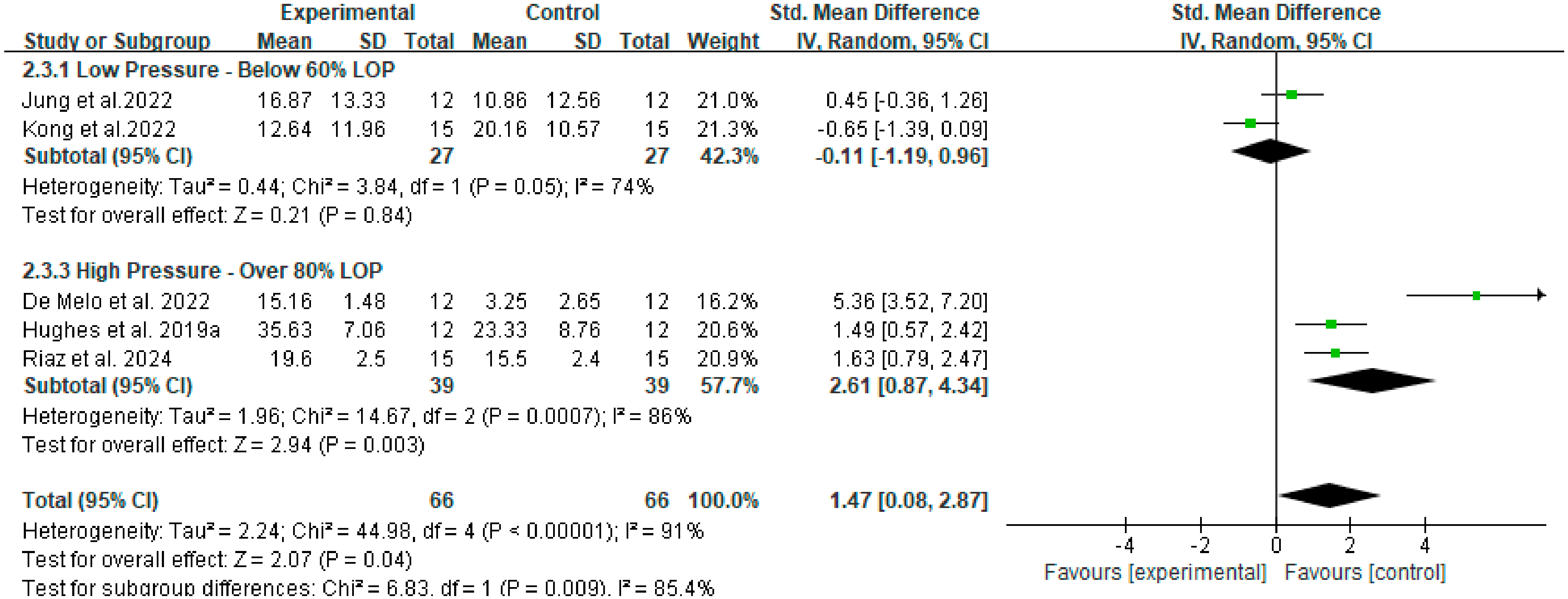

Figure S7. Subgroup analysis of IKDC score according to blood flow restriction (BFR) pressure

Study or Subgroup	Experimental Mean	Experimental SD	Experimental Total	Control Mean	Control SD	Control Total	Weight	Std. Mean Difference IV, Random, 95% CI
**4.2.2 Moderate Term - 4-8 Weeks**								
De Melo et al. 2022	15	3.12	12	-7.83	4.35	12	13.2%	5.82 [3.85, 7.79]
Hughes et al. 2019a	39.75	11.74	12	22	7.48	12	17.1%	1.74 [0.78, 2.71]
Hughes et al. 2019b	-2.05	0.96	12	-1.14	1.64	12	17.5%	-0.65 [-1.48, 0.17]
**Subtotal (95% CI)**			**36**			**36**	**47.8%**	**2.17 [-0.77, 5.10]**
Heterogeneity: Tau² = 6.28; Chi² = 40.47, df = 2 (P < 0.00001); I² = 95%								
Test for overall effect: Z = 1.44 (P = 0.15)								
**4.2.3 Long Term - More Than 8 Weeks**								
De Melo et al. 2022	15.5	3.33	12	3.25	4.04	12	16.0%	3.19 [1.92, 4.47]
Erickson et al. 2024	-4.64	2.13	23	-5.87	1.92	25	18.2%	0.60 [0.02, 1.18]
Khalil et al.2023	-4.35	1.47	18	-3.85	1.28	18	18.0%	-0.35 [-1.01, 0.30]
**Subtotal (95% CI)**			**53**			**55**	**52.2%**	**1.04 [-0.50, 2.58]**
Heterogeneity: Tau² = 1.66; Chi² = 23.82, df = 2 (P < 0.00001); I² = 92%								
Test for overall effect: Z = 1.32 (P = 0.19)								
**Total (95% CI)**			**89**			**91**	**100.0%**	**1.51 [0.20, 2.82]**
Heterogeneity: Tau² = 2.36; Chi² = 65.25, df = 5 (P < 0.00001); I² = 92%								
Test for overall effect: Z = 2.26 (P = 0.02)								
Test for subgroup differences: Chi² = 0.44, df = 1 (P = 0.50), I² = 0%								

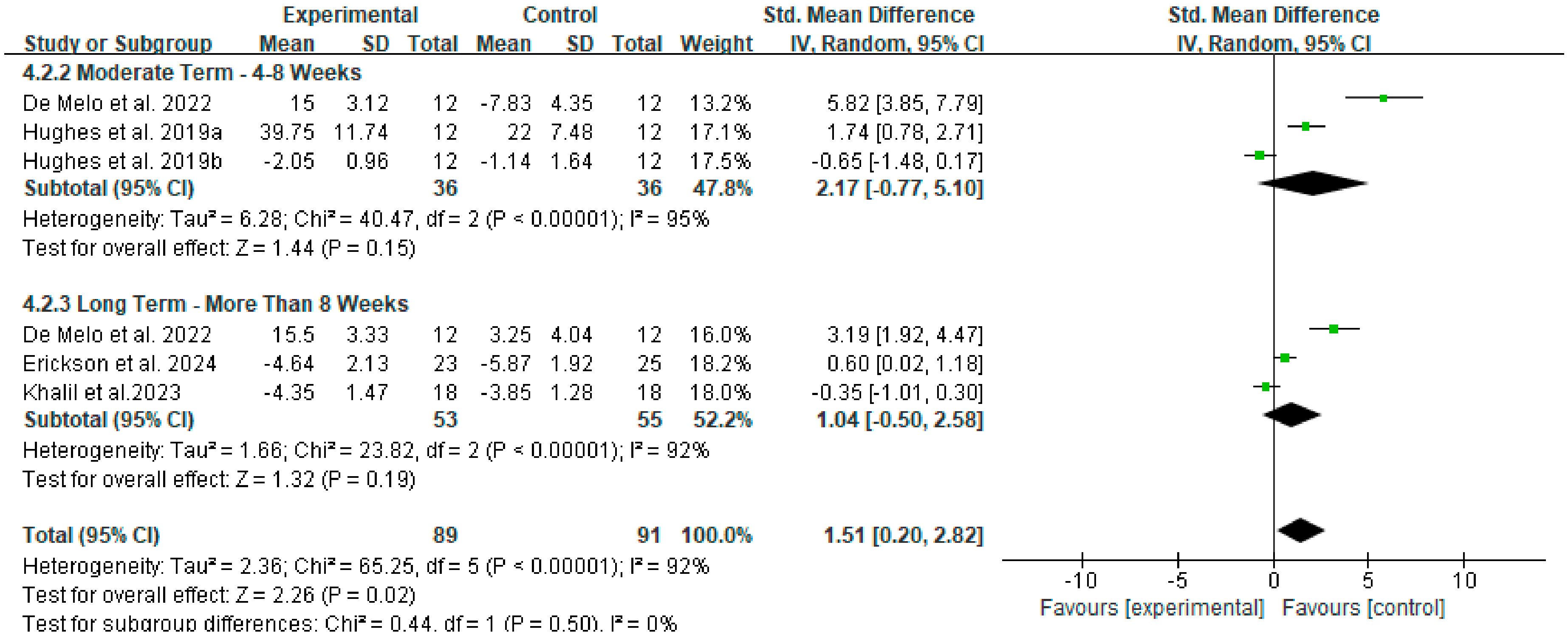

Figure S8. Subgroup analysis of pain according to intervention duration

Study or Subgroup	Experimental Mean	Experimental SD	Experimental Total	Control Mean	Control SD	Control Total	Weight	Std. Mean Difference IV, Random, 95% CI
**5.1.3 Balance test**								
Erickson et al. 2024	-4.64	2.13	23	-5.87	1.92	25	20.5%	0.60 [0.02, 1.18]
Huang et al.2023	-9.51	2.28	41	-4.58	3.2	41	20.7%	-1.76 [-2.27, -1.24]
Hughes et al. 2019a	59	22	12	33	19	12	19.2%	1.22 [0.34, 2.11]
Jung et al.2022	8.14	8.23	12	3.81	8.01	12	19.6%	0.51 [-0.30, 1.33]
Kong et al.2022	7.09	6.47	15	2.92	6.63	15	19.9%	0.62 [-0.12, 1.35]
**Subtotal (95% CI)**			**103**			**105**	**100.0%**	**0.22 [-0.96, 1.40]**
Heterogeneity: Tau² = 1.67; Chi² = 60.04, df = 4 (P < 0.00001); I² = 93%								
Test for overall effect: Z = 0.36 (P = 0.72)								
**Total (95% CI)**			**103**			**105**	**100.0%**	**0.22 [-0.96, 1.40]**
Heterogeneity: Tau² = 1.67; Chi² = 60.04, df = 4 (P < 0.00001); I² = 93%								
Test for overall effect: Z = 0.36 (P = 0.72)								
Test for subgroup differences: Not applicable								

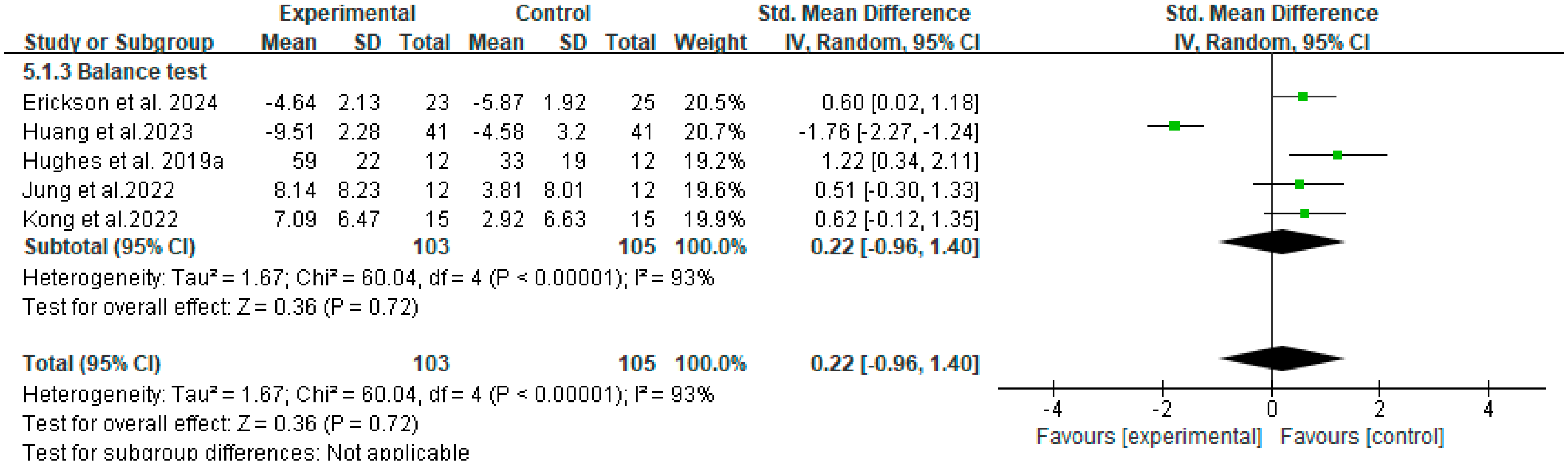

Figure S9. Overall effect of balance

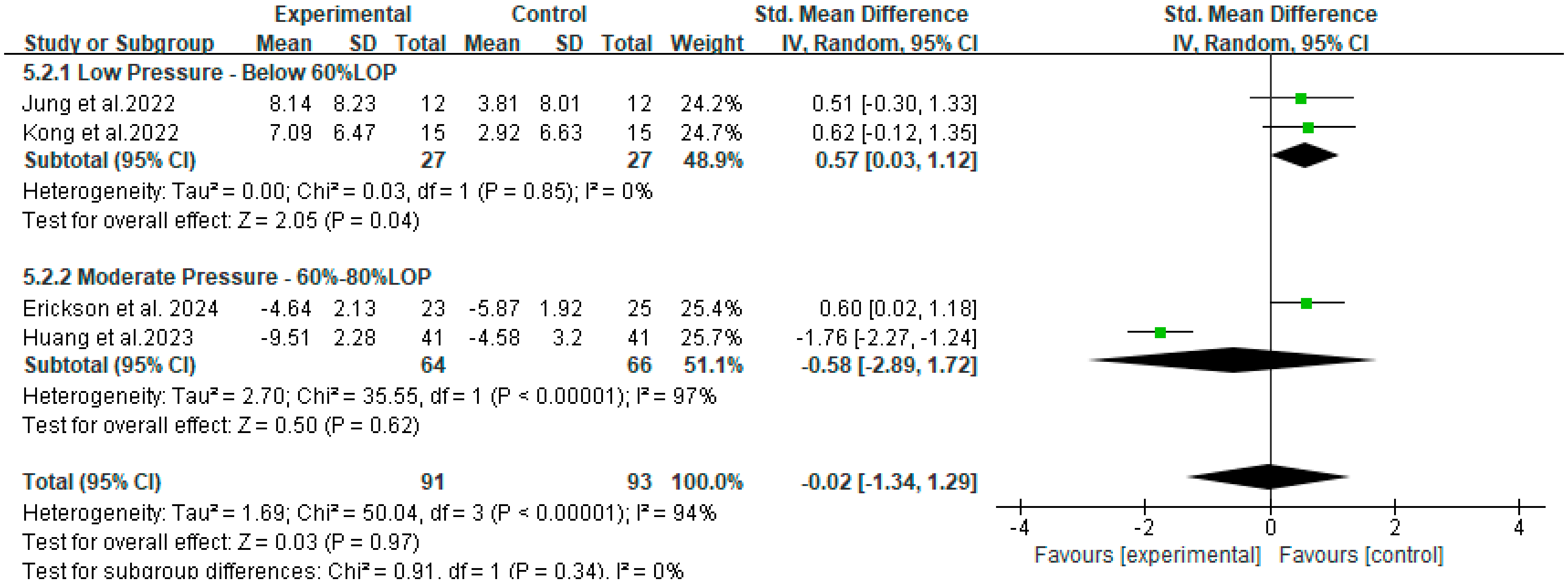

Figure S10. Subgroup analysis of balance according to blood flow restriction (BFR) pressure

Study or Subgroup	Experimental Mean	Experimental SD	Experimental Total	Control Mean	Control SD	Control Total	Weight	Std. Mean Difference IV, Random, 95% CI
**2.6.1 Moderate Term - 4-8 Weeks**								
Huang et al.2023	-9.51	2.28	41	-4.58	3.2	41	20.7%	-1.76 [-2.27, -1.24]
Hughes et al. 2019a	59	22	12	33	19	12	19.2%	1.22 [0.34, 2.11]
**Subtotal (95% CI)**			**53**			**53**	**40.0%**	**-0.29 [-3.21, 2.63]**
Heterogeneity: Tau² = 4.30; Chi² = 32.58, df = 1 (P < 0.00001); I² = 97%								
Test for overall effect: Z = 0.20 (P = 0.85)								
**2.6.2 Long Term - More Than 8 Weeks**								
Erickson et al. 2024	-4.64	2.13	23	-5.87	1.92	25	20.5%	0.60 [0.02, 1.18]
Jung et al.2022	8.14	8.23	12	3.81	8.01	12	19.6%	0.51 [-0.30, 1.33]
Kong et al.2022	7.09	6.47	15	2.92	6.63	15	19.9%	0.62 [-0.12, 1.35]
**Subtotal (95% CI)**			**50**			**52**	**60.0%**	**0.58 [0.19, 0.98]**
Heterogeneity: Tau² = 0.00; Chi² = 0.04, df = 2 (P = 0.98); I² = 0%								
Test for overall effect: Z = 2.88 (P = 0.004)								
**Total (95% CI)**			**103**			**105**	**100.0%**	**0.22 [-0.96, 1.40]**
Heterogeneity: Tau² = 1.67; Chi² = 60.04, df = 4 (P < 0.00001); I² = 93%								
Test for overall effect: Z = 0.36 (P = 0.72)								
Test for subgroup differences: Chi² = 0.34, df = 1 (P = 0.56), I² = 0%								

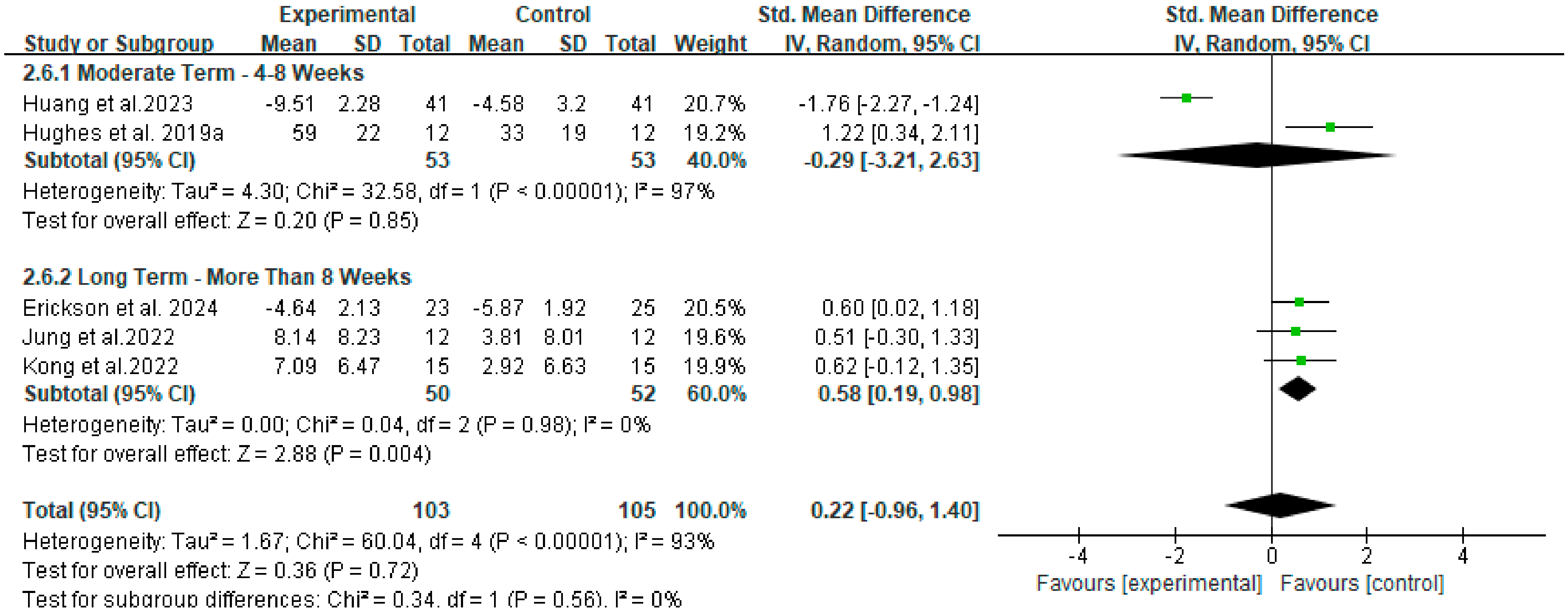

Figure S11. Subgroup analysis of balance according to intervention duration

Table S1. Search strategies used across electronic databases

Database	Search Strategy*	Records Retrieved (n)
PubMed	("blood flow restriction" OR BFR OR KAATSU OR "vascular occlusion training" OR "occlusion training" OR "ischemic training" OR "ischemia training") AND ("anterior cruciate ligament reconstruction" OR ACLR OR knee rehabilitation OR knee surgery OR meniscectomy OR "meniscus repair" OR "total knee arthroplasty" OR TKA) NOT (systematic review OR meta-analysis OR review)	86
Embase	Blood flow restriction terms AND knee surgery/rehabilitation terms NOT review articles	276
Cochrane CENTRAL	Blood flow restriction terms AND knee surgery/rehabilitation terms	1,604
EBSCOhost (all databases)	Blood flow restriction terms AND knee surgery/rehabilitation terms NOT review articles	131
Scopus	TITLE-ABS-KEY (blood flow restriction terms) AND TITLE-ABS-KEY (knee surgery/rehabilitation terms) NOT review articles	119
Web of Science Core Collection	Topic search: blood flow restriction terms AND knee surgery/rehabilitation terms NOT review articles	96
Sage Journals Online	Blood flow restriction terms AND knee surgery/rehabilitation terms	—
ScienceDirect	Blood flow restriction terms AND knee surgery/rehabilitation terms	23
SpringerLink	Blood flow restriction terms AND knee surgery/rehabilitation terms	9
Taylor & Francis Online	Blood flow restriction terms AND knee surgery/rehabilitation terms NOT review articles	51

*The search strategy combined two primary concepts:

(1) blood flow restriction training ("blood flow restriction", BFR, KAATSU, vascular occlusion training, occlusion training, ischemic training, ischemia training) and

(2) knee surgery and rehabilitation-related conditions (anterior cruciate ligament reconstruction, ACLR, knee rehabilitation, knee surgery, meniscectomy, meniscus repair, total knee arthroplasty, TKA).

Database-specific controlled vocabulary (e.g., MeSH and Emtree terms) was incorporated when available. Searches were adapted to the syntax requirements of each database.

Table S2. Certainty of Evidence Assessment Using the GRADE Approach

Outcome	No. of Studies	Risk of Bias	Inconsistency	Indirectness	Imprecision	BFR (n)	Control (n)	Effect Estimate (SMD, 95% CI)	Certainty of Evidence
Muscle strength	9	Serious	Not serious	Not serious	Not serious	164	164	0.80 (0.56 to 1.03)	⊕⊕⊕◯ Moderate[a]
Function	6	Serious	Serious	Not serious	Not serious	107	107	1.21 (0.51 to 1.91)	⊕⊕◯◯ Low[ab]
Balance	5	Serious	Serious	Not serious	Serious	103	105	0.22 (−0.96 to 1.40)	⊕◯◯◯ Very low[abc]
Muscle morphology	6	Serious	Serious	Not serious	Serious	165	165	−0.33 (−1.00 to 0.34)	⊕◯◯◯ Very low[abc]
Pain and symptoms	5	Serious	Serious	Not serious	Serious	77	79	0.82 (−0.28 to 1.92)	⊕◯◯◯ Very low[abc]

Abbreviations: BFR, blood flow restriction; CI, confidence interval; SMD, standardized mean difference.

Explanations:

[a] Most included studies did not adequately report allocation concealment or blinding of participants and personnel.

[b] Substantial statistical heterogeneity was observed across studies. Subgroup analyses based on intervention duration did not adequately explain the observed heterogeneity.

[c] Serious imprecision was present because of the limited total sample size and wide confidence intervals.

Table S3. PRISMA checklist

Section and Topic	Item #	Checklist item	Location where item is reported
TITLE			
Title	1	Identify the report as a systematic review.	Title
ABSTRACT			
Abstract	2	See the PRISMA 2020 for Abstracts checklist.	Abstract
INTRODUCTION			
Rationale	3	Describe the rationale for the review in the context of existing knowledge.	Introduction
Objectives	4	Provide an explicit statement of the objective(s) or question(s) the review addresses.	Introduction
METHODS			
Eligibility criteria	5	Specify the inclusion and exclusion criteria for the review and how studies were grouped for the syntheses.	Eligibility Criteria
Information sources	6	Specify all databases, registers, websites, organisations, reference lists and other sources searched or consulted to identify studies. Specify the date when each source was last searched or consulted.	Data Sources and Search Strategy
Search strategy	7	Present the full search strategies for all databases, registers and websites, including any filters and limits used.	Supplementary
Selection process	8	Specify the methods used to decide whether a study met the inclusion criteria of the review, including how many reviewers screened each record and each report retrieved, whether they worked independently, and if applicable, details of automation tools used in the process.	Study Selection and Screening Process
Data collection	9	Specify the methods used to collect data from reports, including how many reviewers collected data from each report,	Data extraction

Section and Topic	Item #	Checklist item	Location where item is reported
process		whether they worked independently, any processes for obtaining or confirming data from study investigators, and if applicable, details of automation tools used in the process.	
Data items	10a	List and define all outcomes for which data were sought. Specify whether all results that were compatible with each outcome domain in each study were sought (e.g. for all measures, time points, analyses), and if not, the methods used to decide which results to collect.	Data extraction
	10b	List and define all other variables for which data were sought (e.g. participant and intervention characteristics, funding sources). Describe any assumptions made about any missing or unclear information.	Data extraction
Study risk of bias assessment	11	Specify the methods used to assess risk of bias in the included studies, including details of the tool(s) used, how many reviewers assessed each study and whether they worked independently, and if applicable, details of automation tools used in the process.	Quality Assessment and Risk of Bias
Effect measures	12	Specify for each outcome the effect measure(s) (e.g. risk ratio, mean difference) used in the synthesis or presentation of results.	Data extraction
Synthesis methods	13a	Describe the processes used to decide which studies were eligible for each synthesis (e.g. tabulating the study intervention characteristics and comparing against the planned groups for each synthesis (item #5)).	Data Synthesis
	13b	Describe any methods required to prepare the data for presentation or synthesis, such as handling of missing summary statistics, or data conversions.	Statistical analysis
	13c	Describe any methods used to tabulate or visually display results of individual studies and syntheses.	Statistical analysis
	13d	Describe any methods used to synthesize results and provide a rationale for the choice(s). If meta-analysis was performed,	Statistical

Section and Topic	Item #	Checklist item	Location where item is reported
		describe the model(s), method(s) to identify the presence and extent of statistical heterogeneity, and software package(s) used.	analysis
	13e	Describe any methods used to explore possible causes of heterogeneity among study results (e.g. subgroup analysis, meta-regression).	Statistical analysis
	13f	Describe any sensitivity analyses conducted to assess robustness of the synthesized results.	Statistical analysis
Reporting bias assessment	14	Describe any methods used to assess risk of bias due to missing results in a synthesis (arising from reporting biases).	Statistical analysis
Certainty assessment	15	Describe any methods used to assess certainty (or confidence) in the body of evidence for an outcome.	Quality Assessment and Risk of Bias
RESULTS			
Study selection	16a	Describe the results of the search and selection process, from the number of records identified in the search to the number of studies included in the review, ideally using a flow diagram.	Fig.1 Prisma flow chart
	16b	Cite studies that might appear to meet the inclusion criteria, but which were excluded, and explain why they were excluded.	Fig.1 Prisma flow chart
Study characteristics	17	Cite each included study and present its characteristics.	Table 1
Risk of bias in	18	Present assessments of risk of bias for each included study.	Fig. 2

Section and Topic	Item #	Checklist item	Location where item is reported
studies			
Results of individual studies	19	For all outcomes, present, for each study: (a) summary statistics for each group (where appropriate) and (b) an effect estimate and its precision (e.g. confidence/credible interval), ideally using structured tables or plots.	Table 1 and forest plots
Results of syntheses	20a	For each synthesis, briefly summarise the characteristics and risk of bias among contributing studies.	Result, basic information of included studies
	20b	Present results of all statistical syntheses conducted. If meta-analysis was done, present for each the summary estimate and its precision (e.g. confidence/credible interval) and measures of statistical heterogeneity. If comparing groups, describe the direction of the effect.	Result
	20c	Present results of all investigations of possible causes of heterogeneity among study results.	Result
	20d	Present results of all sensitivity analyses conducted to assess the robustness of the synthesized results.	Result
Reporting biases	21	Present assessments of risk of bias due to missing results (arising from reporting biases) for each synthesis assessed.	Statistical analysis
Certainty of evidence	22	Present assessments of certainty (or confidence) in the body of evidence for each outcome assessed.	Result
DISCUSSION			
Discussion	23a	Provide a general interpretation of the results in the context of other evidence.	Discussion
	23b	Discuss any limitations of the evidence included in the review.	Limitation

Section and Topic	Item #	Checklist item	Location where item is reported
	23c	Discuss any limitations of the review processes used.	Limitation
	23d	Discuss implications of the results for practice, policy, and future research.	Practical Application and suggestions
OTHER INFORMATION			
Registration and protocol	24a	Provide registration information for the review, including register name and registration number, or state that the review was not registered.	Study Design and Protocol Registration
	24b	Indicate where the review protocol can be accessed, or state that a protocol was not prepared.	Study Design and Protocol Registration
	24c	Describe and explain any amendments to information provided at registration or in the protocol.	Study Design and Protocol Registration
Support	25	Describe sources of financial or non-financial support for the review, and the role of the funders or sponsors in the review.	Funding/ financial support
Competing interests	26	Declare any competing interests of review authors.	Conflict of interest

Section and Topic	Item #	Checklist item	Location where item is reported
			declaration
Availability of data, code and other materials	27	Report which of the following are publicly available and where they can be found template data collection forms; data extracted from included studies; data used for all analyses; analytic code; any other materials used in the review.	Supplementary

From: Page MJ, McKenzie JE, Bossuyt PM, Boutron I, Hoffmann TC, Mulrow CD, et al. The PRISMA 2020 statement: an updated guideline for reporting systematic reviews. BMJ 2021;372: n71. https://doi.org/10.1136/bmj.n71

This work is licensed under CC BY 4.0. To view a copy of this license, visit https://creativecommons.org/licenses/by/4.0/

Table S4. PRISMA abstract checklist

Section and Topic	Item #	Checklist item	Reported (Yes/No)
TITLE			
Title	1	Identify the report as a systematic review.	Yes
BACKGROUND			
Objectives	2	Provide an explicit statement of the main objective(s) or question(s) the review addresses.	Yes
METHODS			
Eligibility criteria	3	Specify the inclusion and exclusion criteria for the review.	Yes
Information sources	4	Specify the information sources (e.g. databases, registers) used to identify studies and the date when each was last searched.	Yes
Risk of bias	5	Specify the methods used to assess risk of bias in the included studies.	Yes
Synthesis of results	6	Specify the methods used to present and synthesise results.	Yes
RESULTS			
Included studies	7	Give the total number of included studies and participants and summarise relevant characteristics of studies.	Yes
Synthesis of results	8	Present results for main outcomes, preferably indicating the number of included studies and participants for each. If meta-analysis was done, report the summary estimate and confidence/credible interval. If comparing groups, indicate the direction of the effect (i.e. which group is favoured).	Yes
DISCUSSION			
Limitations of evidence	9	Provide a brief summary of the limitations of the evidence included in the review (e.g. study risk of bias, inconsistency and imprecision).	Yes
Interpretation	10	Provide a general interpretation of the results and important implications.	Yes

Section and Topic	Item #	Checklist item	Reported (Yes/No)
OTHER			
Funding	11	Specify the primary source of funding for the review.	Yes
Registration	12	Provide the register name and registration number.	Yes

From: Page MJ, McKenzie JE, Bossuyt PM, Boutron I, Hoffmann TC, Mulrow CD, et al. The PRISMA 2020 statement: an updated guideline for reporting systematic reviews. BMJ 2021;372: n71. https://doi.org/10.1136/bmj.n71

This work is licensed under CC BY 4.0. To view a copy of this license, visit https://creativecommons.org/licenses/by/4.0/
